# Fluctuations, Correlations and the Estimation of Concentrations inside Cells

**DOI:** 10.1371/journal.pone.0151132

**Published:** 2016-03-10

**Authors:** Emiliano Pérez Ipiña, Silvina Ponce Dawson

**Affiliations:** Departamento de Física, FCEN-UBA, and IFIBA, CONICET, Ciudad Universitaria, Pabellón I, (1428) Buenos Aires, Argentina; University of Milano-Bicocca, ITALY

## Abstract

Information transmission in cells occurs quite accurately even when concentration changes are “read” by individual binding sites. In this paper we study ligand number and site occupancy fluctuations when ligands diffuse and react going beyond the analyses that focus on their asymptotic decay. In this way we show that, for immobile binding sites, fluctuations in the number of bound molecules decay on a relatively fast scale before the asymptotic behavior kicks in. This result can explain the observed co-existence of highly fluctuating instantaneous transcriptional activities with accumulated mRNA concentrations that have relatively small noise levels. We also show that the initial stages of the decay in the bound molecule number fluctuations have one or two characteristic timescales depending on the concentration of free molecules. This transition can explain the changes in enzyme activity observed at the single molecule level.

## Introduction

The transmission of information in cells usually involves changes in concentration that are “read” by target molecules. This occurs in a fluctuating environment. Yet cells respond quite reliably to various changes [1, 2]. The accuracy of the reading mechanism is key in the case of morphogens, molecules whose non-uniform distribution results in cell differentiation [3]. Most often this patterning process involves the binding of transcription factors to sites on DNA controlling the levels of protein production. The relationship between the concentration of a protein and of the transcription factor that regulates its production depends on various binding processes. How faithful the spatial distribution of protein concentration reflects that of the transcription factor depends on how the concentration of the latter is read by the binding sites. This relationship has been studied during the early stages of development of *Drosophila melanogaster* embryos. The analysis of the variability of the concentrations of the protein Hunchback (Hb) and of the transcription factor Bicoid (Bcd) involved in its production shows that the resulting pattern is compatible with detecting [Bcd] with a 10% error [3]. Considering the random arrivals of individual Bcd molecules to a small neighborhood around a putative DNA binding site the calculations of [3] concluded that only after a long time (∼2*h*) compared to the embryo developmental time [Bcd] could be inferred with this precision. In [3] a spatial averaging between neighboring nuclei was invoked to reconcile this computation with the observations. Some sort of spatial averaging was also invoked to explain recent observations of transcriptional regulation in *Drosophila melanogaster* embryos [4, 5] where the instantaneous production of mRNA varied by up to 50% between loci of transcription but the cytoplasmic mRNA accumulated around a locus fluctuated by less than 8%. This level of noise reduction could not be accounted for solely by time averaging. The existence of some type of spatial averaging is possible during the early stages of *Drosophila* development because several nuclei share a common cytoplasm. Thus, it is relevant to understand how fluctuations in the accumulated number of product molecules relate to those of the molecules that regulate their production when there are several production sites. In other words, how faithfully ligand concentration can be inferred by binding sites when there are several of them competing for the same ligands. In this paper we address this point.

The seminal work of Berg and Purcell [6] showed that the time it takes for a ligand concentration to be estimated by binding sites with a certain accuracy depends on the diffusion coefficient of the ligand. The subsequent studies of [1, 7] extended this work finding that ligand diffusion imposed a fundamental limit on accuracy. The estimate of ∼2*hs* for the concentration of Bcd to be read with a 10% accuracy in *Drosophila melanogaster* embryos (with no spatial averaging) [3] was derived using the Bcd diffusion coefficient determined in Fluorescence Recovery After Photobleaching (FRAP) experiments [8]. This diffusion coefficient was estimated to be an order of magnitude larger using Fluorescence Correlation Spectroscopy [9]. These two apparently disparate estimates have recently been shown to be compatible [10] if they are assumed to correspond to the two effective diffusion coefficients that describe the transport of molecules that diffuse and react [11]. In fact, Bcd, being a transcription factor, diffuses and reacts at least with putative binding sites on DNA. If, as in the case of Bcd in *Drosophila* embryos, several binding sites compete for the same pool of Bcd molecules, what is the diffusion coefficient that sets the limit for the precision with which [Bcd] can be read by those binding sites? This is one motivating question of our paper.

The problem of how accurately a ligand concentration can be inferred [12] was recently re-analyzed for the case of a single binding site [7]. Building upon previous studies on diffusion-limited reactions [13, 14] the work of [7] derives an expression for the mean (asymptotic) correlation time that characterizes fluctuations in the probability that the site be bound to a ligand. The authors validate their expression, which differs slightly from the one obtained in [1], via numerical simulations. As we discuss later, the main difference between the results of [7] and [1] may be attributed to different linearizations of the non-linear problem that rules the dynamics. The quantity of interest in these works is the fraction of time, *f*_*b*_(*T*_*obs*_), that the binding site spends bound during an “interaction” interval, [0, *T*_*obs*_], and how it differs from the equilibrium probability, *p*_*b*_, that it be bound given the actual concentration of ligand. Thus, introducing the stochastic variable, *N*^(*b*)^(*t*), such that *N*^(*b*)^ = 1, if the site is bound and 0, otherwise, *f*_*b*_(*T*_*obs*_) is equal to the average:

$${\bar{N}}^{\left(b\right)}\left(T_{obs}\right)\equiv \frac{1}{T_{obs}}\int_{0}^{T_{obs}}dtN^{\left(b\right)}\left(t\right),$$ (1)

and *p*_*b*_ is equal to the mean, $\langle {\bar{N}}^{\left(b\right)}\rangle =\langle N^{\left(b\right)}\rangle$. In the case of more than one binding site, *f*_*b*_(*T*_*obs*_) and *p*_*b*_ are defined similarly but divided by the total number of sites, *N*_*ST*_. The squared difference between them is estimated as

$$\mathrm{var}\left({\bar{N}}^{\left(b\right)}\left(T_{obs}\right)\right)\equiv \langle {\left({\bar{N}}^{\left(b\right)}\left(T_{obs}\right)-\langle N^{\left(b\right)}\rangle \right)}^{2}\rangle ,$$ (2)

and the relative error as:

$$\Delta_{r}\left({\bar{N}}^{\left(b\right)}\right)\equiv {\left(\mathrm{var}\left({\bar{N}}^{\left(b\right)}\left(T_{obs}\right)\right)\right)}^{1/2}/\langle N^{\left(b\right)}\rangle .$$ (3)

The variance in Eq (2) is related [12] to the autocorrelation function (ACF),

$$G^{\left(b\right)}\left(\tau \right)=\langle \left(N^{\left(b\right)}\left(t\right)-\langle N^{\left(b\right)}\rangle \right)\left(N^{\left(b\right)}\left(t+\tau \right)-\langle N^{\left(b\right)}\rangle \right)\rangle ,$$ (4)

by:

$$\mathrm{var}\left({\bar{N}}^{\left(b\right)}\left(T_{obs}\right)\right)=\frac{1}{T_{obs}^{2}}\int_{0}^{T_{obs}}dt^{\prime }\int_{0}^{T_{obs}}dtG^{\left(b\right)}\left(t^{\prime }-t\right).$$ (5)

Thus, the correlation times, *τ*_*i*_, of the ACF, *G*^(*b*)^, rule the decay of the relative error with time. The studies of [1, 7, 12] focus on the asymptotic decay of this error. Introducing the mean (asymptotic) correlation time [12]:

$$\tau^{\left(b\right)}\equiv \frac{1}{\mathrm{var}(N^{\left(b\right)})}\int_{0}^{\infty }d\tau G^{\left(b\right)}\left(\tau \right),$$ (6)

it can be shown that, if *T*_*obs*_ ≫ max_*i*_ *τ*_*i*_, it is [12]:

$${\left(\Delta_{r}\left({\bar{N}}^{\left(b\right)}\right)\right)}^{2}\approx \frac{2\tau^{\left(b\right)}}{T_{obs}}\frac{\mathrm{var}(N^{\left(b\right)})}{{\langle N^{\left(b\right)}\rangle }^{2}}.$$ (7)

In the present paper, we do not limit the analyses to this asymptotic behavior, but rather look at how this behavior is achieved. It is clear from Eq (7) that the error depends on var(*N*^(*b*)^). In the case at hand var(*N*^(*b*)^) depends on the mean number of free ligand molecules that are within an interaction distance of the binding site. The number of free ligands in this interaction volume is a (highly fluctuating) random variable. The problem is nonlinear and complicated and the derivation of Eq (7) assumes that the system is close to equilibrium. In the present paper we derive an approximated expression for the relative error, Eq (3), that considers the time it takes for the number of free ligands in the interaction volume to approach its mean value. This correction can explain the observed co-existence of highly fluctuating instantaneous transcriptional activities and of accumulated mRNA concentrations with relatively low noise [4, 5]. Our studies show that it is possible to interpret the “high” noise reduction observed in *Drosophila melanogaster* embryos with some type of spatial averaging: not that of the “product” (the mRNA), as suggested in [4], but that of the “substrate” or ligand (the transcription factor). Another difference of our work with respect to previous ones is that we not only look at fluctuations in the occupation state of single binding sites but of groups of them. In this way we can analyze some results that come from experiments of very good resolution but not high enough to allow the direct observation of individual sites at work [4].

In order to study the decay of the error before the asymptotic behavior is reached, we build upon our previous works on the analysis of optical experiments when molecules diffuse and react [15–17]. We derive analytic expressions for the relevant ACFs in certain limits. In this way we obtain the individual correlation times that eventually contribute to the mean (asymptotic) time defined in Eq (6). Although some real situations may not fit within any of the two limits, knowing the behavior for both of them gives an indication of what may happen in between. One of the limits, on the other hand, always holds for sufficiently long lag times [17]. Having analytic expressions for the ACF not only allows us to study the “early” decay of the relative error in the estimated number of bound or free molecules in a volume. It also lets us look at other properties of processes that involve binding when observed at the single molecule level. In particular, using our analytic ACFs we derive an approximated dwell-time distribution between individual bindings whose time dependence may be studied as a function of parameters. This provides a tool to interpret the changes in the dwell-time distribution between individual enzyme turnovers observed in [18], where the catalytic actitivity of *β*-galactosidase was tested at the single-molecule level. In [18] the transition of the distribution from a mono to a multi-exponential function with increasing substrate concentration was attributed to fluctuations in the substrate-enzyme binding/unbinding rate constants. Our studies show that the observed changes can be due to a transition from a situation in which the correlation time is dominated by the reactions to another in which it is dominated by diffusion. While in the former case the distribution is mono-exponential, in the latter it decays as a rational function of time that looks multi-exponential.

## Materials and Methods

### The model

We consider a system of particles (*e.g.*, transcription factors or substrate molecules), *P*^(*f*)^, that diffuse with (free) coefficient, *D*_*f*_, and react with binding sites, *S*, according to [11, 16, 17]:

$$P^{\left(f\right)}+S\;\overset{k_{on}}{\underset{k_{off}}{⇄}}P^{\left(b\right)}.$$ (8)

We assume that the binding sites diffuse with coefficient *D*_*S*_ ≪ *D*_*f*_ (in all the examples we consider *D*_*S*_ = 0) and that *S* is so massive that the free coefficient of *P*^(*b*)^ is *D*_*S*_ too. We consider a total volume, *V*_*T*_, over which the molecules diffuse and the concentrations, [*P*^(*f*)^], [*P*^(*b*)^], [*S*], are approximately constant, uniform and in equilibrium among themselves ([*P*^(*f*)^][*S*] = *K*_*D*_[*P*^(*b*)^]), and an observation volume, *V*_*obs*_, where the number of molecules, *N*^(*f*)^, *N*^(*b*)^ and *N*^(*S*)^, are counted. The means of these stochastic variables satisfy 〈*N*^(*f*)^〉 = [*P*^(*f*)^]*V*_*obs*_, 〈*N*^(*b*)^〉 = [*P*^(*b*)^]*V*_*obs*_ and 〈*N*^(*S*)^〉 = [*S*]*V*_*obs*_ if *D*_*s*_ ≠ 0. If *D*_*S*_ = 0 and *V*_*obs*_ ≪ *V*_*T*_, there could be a local equilibrium in *V*_*obs*_ slightly different from the one in *V*_*T*_ that depends on the (fixed) total number of binding sites in *V*_*obs*_, *N*_*ST*_ ≡ *N*^(*b*)^+*N*^(*S*)^. The aim is to determine the difference between the mean and the average, ${\bar{N}}^{\left(s\right)}\left(T_{obs}\right)=\frac{1}{T_{obs}}\int_{0}^{T_{obs}}dtN^{\left(s\right)}$ of each stochastic variable (*s* = *f*, *b*, *S*) after an observation time, *T*_*obs*_. These differences reflect by how much the concentrations that can be estimated after a time, *T*_*obs*_, by counting how many particles of each species are, on average, in the observation volume, *V*_*obs*_, differ from the bulk concentrations in the total volume, *V*_*T*_, which are proportional to the mean of the number of particles of each species. We estimate these differences using Eq (2) replacing *N*^(*b*)^ by the corresponding *N*^(*s*)^ in each case. Equivalently, we compute the variances using Eqs (4) and (5), replacing, in both cases, *b* by the corresponding *s*. More details about the model and the calculations that are described in what follows can be found in S1 Text.

### ACF and mean correlation time

For the analytic calculations we compute the ACF as in the case of FCS experiments [16, 17, 19]. We briefly describe here the main steps. A more detailed description is provided in S1 Text. Instead of adding all the particles of species (*s*) in *V*_*obs*_ to compute *N*^(*s*)^, we add all the particles of species (*s*) in *V*_*T*_ but with a Gaussian weight: $N^{\left(s\right)}=\int_{V_{T}}d^{3}\vec{r}I\left(\vec{r}\right)c^{\left(s\right)}$ where $I\left(\vec{r}\right)=\exp(-\frac{r^{2}}{2a^{2}})$, $r=|\vec{r}|$, *a* is half the waist of the Gaussian and $c^{\left(s\right)}=\sum_{i_{s}}\delta \left(\vec{r}-{\vec{r}}_{i_{s}}\left(t\right)\right)$ with the sum running over all the molecules of species (*s*) and ${\vec{r}}_{i_{s}}\left(t\right)$ the location of each of them at time *t*. In this way, it is $V_{obs}=\int d^{3}\vec{r}I\left(\vec{r}\right)=8\pi^{3/2}a^{3}$ and:

$$G^{\left(s\right)}\left(\tau \right)=\int d\vec{r}\int d\vec{r}{\;}^{\prime }\;\langle \delta c^{\left(s\right)}\left(\vec{r},0\right)\delta c^{\left(s\right)}\left(\vec{r}{\;}^{\prime },\tau \right)\rangle ,$$ (9)

where $\delta c^{\left(s\right)}\equiv c^{\left(s\right)}\left(\vec{r},t\right)-\langle N^{\left(s\right)}\rangle /V_{obs}$. As done in [15, 19], for the analytic computation of *G*^(*s*)^(*τ*) we calculate the differences, *δc*^(*s*)^, for the 3 species of the system, as the solution of a linearized version of the reaction-diffusion equations that describe the dynamics of the concentrations of *P*^(*f*)^, *P*^(*b*)^ and *S*. As described in S1 Text, there are two possible linearizations that lead to two different expressions of the asymptotic correlation time (the one obtained in [7] and the one obtained in [1]). The steps that are followed to proceed with the computations are equivalent in both cases, but the final results differ from one another. In order to obtain the correlation times we express Eq (9) in terms of the (branches of) eigenvalues and eigenvectors of the linearized reaction-diffusion equations in Fourier space:

$$G^{\left(s\right)}\left(\tau \right)=\frac{1}{{\left(2\pi \right)}^{3}}\int d\vec{\xi }{\left(\hat{I}\left(\vec{\xi }\right)\right)}^{2}\sum_{m}X_{j}^{\left(m\right)}\exp\left(\lambda^{\left(m\right)}\tau \right){\left(X^{-1}\sigma^{2}\right)}_{j}^{\left(m\right)}$$ (10)

where the subscript, *j*, refers to the species (*j* = 1 for *s* = *f*, and *j* = 2 for *s* = *b*) and the index, (*m*), labels the eigenvalues, $\hat{I}\left(\vec{\xi }\right)$ is the Fourier transform of $I(\vec{r})$ and $\vec{\xi }$ is the conjugate variable of $\vec{r}$, *X* is the matrix of eigenvectors, *λ*^(*m*)^ is the *m*-th eigenvalue and *σ*^2^ is the matrix of initial correlations between the species, $\sigma_{ij}^{2}=\langle \delta N^{\left(s\right)}\left(0\right)\delta N^{\left(s^{\prime }\right)}\left(0\right)\rangle$ with *i*, *j* the indices corresponding to species *s* and *s*′, respectively. As in [15] we assume that $\langle \delta c^{\left(s\right)}\left(\vec{r},t\right)\delta c^{\left(s^{\prime }\right)}\left({\vec{r}}^{\prime },t\right)\rangle =\mathrm{var}\left(N^{\left(s\right)}\right)/V_{obs}\delta_{ij}\delta \left(\vec{r}-{\vec{r}}^{\prime }\right)$ with a Poisson statistics for *s* = *f*, var(*N*^(*f*)^) = 〈*N*^(*f*)^〉, and binomial for *s* = *b*, var(*N*^(*b*)^) = (1 − *p*_*b*_)〈*N*^(*b*)^〉. The mean correlation time, *τ*^(*b*)^, can be computed exactly using Eq (6). It is:

$$\tau^{\left(b\right)}=\frac{p_{b}N_{ST}}{\sqrt{2\pi^{3}}a\left[P^{\left(f\right)}\right]D_{f}}+\frac{1-p_{b}}{k_{off}},$$ (11)

or

$$\tau^{\left(b\right)}=\frac{p_{b}\left(1-p_{b}\right)N_{ST}}{\sqrt{2\pi^{3}}a\left[P^{\left(f\right)}\right]D_{f}}+\frac{1-p_{b}}{k_{off}},$$ (12)

depending on the linearized version of the reaction-diffusion equations that is used to start the computations.

### Approximated ACF in two limits

Even if simple algebraic expressions (Eqs (11) or (12)) are always obtained for the mean (asymptotic) correlation time, *τ*^(*b*)^, we only have Eq (10) for the ACF which is a sum of as many integrals over *ξ* as branches of eigenvalues and eigenvectors of the linearized reaction-diffusion equations in Fourier space. If the eigenvalues are either independent of *ξ* (something that corresponds to an exponential decay in time) or proportional to *ξ*^2^ (something that corresponds to a diffusive decay) the integrals can be performed exactly. Thus there are simple algebraic expressions for the terms (or components) of the ACF that correspond to these types of eigenvalues. As discussed in [16] (see also S1 Text) there are two limits for which all the eigenvalue branches can be expressed as *λ*^(*i*)^ = −*ν*_*i*_ or as −*D*_*i*_ *ξ*^2^ with *ν*_*i*_ a function of the reaction rates and concentrations and *D*_*i*_ depending, in general, on these quantities and on *D*_*f*_. These are the *fast reaction* (*fr*) and the *fast diffusion* limits (*fd*) defined by *τ*_*r*_ ≪ *τ*_*f*_ and *τ*_*f*_ ≪ *τ*_*r*_, respectively with *τ*_*f*_ the diffusion timescale and *τ*_*r*_ the reaction one:

$$\tau_{f}\equiv a^{2}D_{f}^{-1},\;\tau_{r}\equiv {\left(k_{off}+k_{on}\tilde{S}+k_{on}\left[P^{\left(f\right)}\right]\right)}^{-1},$$ (13)

where $\tilde{S}=\left[S_{T}\right]$ in the case of the linearization that leads to Eq (11) and $\tilde{S}=\left[S\right]$ in the case that leads to Eq (12). Given Eqs (11) and (12) we conclude that one of the two terms prevails in the sum that defines *τ*^(*b*)^ in each of these limits.

Approximating Eq (10) in both limits as done in [16] but considering the initial correlations described before [15] (see S1 Text) we obtain simple analytic expressions for the ACF. In the *fd* limit they read:

$$G^{\left(f\right)}\left(\tau \right)=\mathrm{var}\left(N^{\left(f\right)}\right){\left(1+\frac{\left|\tau \right|}{\tau_{f}}\right)}^{-3/2},$$ (14)

$$G^{\left(b\right)}\left(\tau \right)=\mathrm{var}\left(N^{\left(b\right)}\right)e^{-\left|\tau \right|/\tau_{off}},$$ (15)

with *τ*_*f*_ defined in Eq (13) and

$$\tau_{off}^{-1}=\frac{k_{off}}{1-p_{b}}.$$ (16)

In the *fr* limit we obtain:

$$G^{\left(f\right)}\left(\tau \right)=\mathrm{var}\left(N^{\left(f\right)}\right)\left(\frac{{\left(1+\frac{\left|\tau \right|}{\tau_{ef}}\right)}^{-3/2}}{1+\beta }+\frac{\beta e^{-\frac{\left|\tau \right|}{{\tilde{\tau }}_{r}}}}{1+\beta }\right)\;$$ (17)

$$G^{\left(b\right)}\left(\tau \right)=\mathrm{var}\left(N^{\left(b\right)}\right)\left(\frac{\beta {\left(1+\frac{\left|\tau \right|}{\tau_{ef}}\right)}^{-3/2}}{1+\beta }+\frac{e^{-\frac{\left|\tau \right|}{{\tilde{\tau }}_{r}}}}{1+\beta }\right),$$ (18)

with

$$\tau_{ef}\equiv \frac{a^{2}}{D_{ef}}\equiv \frac{a^{2}\left(1+\beta \right)}{D_{f}},\;{\tilde{\tau }}_{r}=\frac{\tau_{off}}{1+\beta }$$ (19)

and *β* = *p*_*b*_ *N*_*ST*_/〈*N*^(*f*)^〉 if we use the linearization that gives Eq (11) and *β* = *p*_*b*_(1 − *p*_*b*_)*N*_*ST*_/〈*N*^(*f*)^〉 if we use the one that leads to Eq (12). The first term in Eqs (17) and (18) corresponds to a diffusive correlation time while the second is reaction dominated.

### Variance and relative errors

We extend Eqs (5) and (3) to any species, *s*, to compute the (square of the) relative error as:

$$\Delta_{r}{\left({\bar{N}}^{\left(s\right)}\right)}^{2}=\frac{\mathrm{var}\left(N^{\left(s\right)}\right)}{T_{obs}^{2}\langle N^{\left(s\right)}\rangle }\int_{0}^{T_{obs}}dt^{\prime }\int_{0}^{T_{obs}}dtG^{\left(s\right)}\left(t^{\prime }-t\right).$$ (20)

Inserting Eqs (14), (15), (17) and (18) into Eq (20) we derive analytic expressions for $\Delta_{r}\left({\bar{N}}^{\left(s\right)}\right)$ in the *fd* and the *fr* limits. We obtain:

$${\left(\Delta_{r}\left({\bar{N}}^{\left(f\right)}\right)\right)}^{2}=\frac{\mathrm{var}\left(N^{\left(f\right)}\right)}{{\langle N^{\left(f\right)}\rangle }^{2}}\frac{4\tau_{f}}{T_{obs}}\left(1+\frac{2\tau_{f}}{T_{obs}}\left(1-{\left(1+\frac{T_{obs}}{\tau_{f}}\right)}^{1/2}\right)\right),$$ (21)

$${\left(\Delta_{r}\left({\bar{N}}^{\left(b\right)}\right)\right)}^{2}=\frac{\mathrm{var}\left(N^{\left(b\right)}\right)}{{\langle N^{\left(b\right)}\rangle }^{2}}\frac{2\tau_{off}}{T_{obs}}\left(1+\frac{\tau_{off}}{T_{obs}}\left(e^{-T_{obs}/\tau_{off}}-1\right)\right),$$ (22)

in the *fd* limit and, in the *fr* one,

$${\left(\Delta_{r}\left({\bar{N}}^{\left(f\right)}\right)\right)}^{2}=\frac{\mathrm{var}\left(N^{\left(f\right)}\right)}{\left(1+\beta \right){\langle N^{\left(f\right)}\rangle }^{2}}\times \left(\frac{2{\tilde{\tau }}_{r}\beta }{T_{obs}}\left(1+\frac{{\tilde{\tau }}_{r}}{T_{obs}}\left(e^{-T_{obs}/{\tilde{\tau }}_{r}}-1\right)\right)+\frac{4\tau_{ef}}{T_{obs}}\left(1+2\frac{\tau_{ef}}{T_{obs}}\left(1-{\left(1+\frac{T_{obs}}{\tau_{ef}}\right)}^{1/2}\right)\right)\right),$$ (23)

$${\left(\Delta_{r}\left({\bar{N}}^{\left(b\right)}\right)\right)}^{2}=\frac{\mathrm{var}\left(N^{\left(b\right)}\right)}{\left(1+\beta \right){\langle N^{\left(b\right)}\rangle }^{2}}\times \left(\frac{2{\tilde{\tau }}_{r}}{T_{obs}}\left(1+\frac{{\tilde{\tau }}_{r}}{T_{obs}}\left(e^{-T_{obs}/{\tilde{\tau }}_{r}}-1\right)\right)+\frac{4\tau_{ef}\beta }{T_{obs}}\left(1+2\frac{\tau_{ef}}{T_{obs}}\left(1-{\left(1+\frac{T_{obs}}{\tau_{ef}}\right)}^{1/2}\right)\right)\right),$$ (24)

For the latter we also work with a simpler expression where we approximate each component of the ACF by a step-wise function (see S1 Text for more details) and obtain

$$\begin{array}{ll} {\left(\Delta_{r}({\bar{N}}^{\left(b\right)})\right)}^{2}= & \frac{\text{var}\left(N^{\left(b\right)}\right)}{(1+\beta ){\langle N^{\left(b\right)}\rangle }^{2}}\times \\ & \;1,\;\text{if}\;T_{obs}\leq {\tilde{\tau }}_{r},\\ & \left(\frac{2{\tilde{\tau }}_{r}}{T_{obs}}\left(1-\frac{{\tilde{\tau }}_{r}}{2T_{obs}}\right)+\beta \right),\;\text{if}\;{\tilde{\tau }}_{r}\leq T_{obs}\leq 2\tau_{ef},\\ & \left(\frac{2{\tilde{\tau }}_{r}}{T_{obs}}\left(1-\frac{{\tilde{\tau }}_{r}}{2T_{obs}}\right)+\beta \frac{4\tau_{ef}}{T_{obs}}\left(1-\frac{2\tau_{ef}}{2T_{obs}}\right)\right),\text{ if}\;T_{obs}\geq 2\tau_{ef}. \end{array}$$ (25)

In some instances we also compute $\Delta_{r}{\left({\bar{N}}^{\left(s\right)}\right)}^{2}$ outside the *fd* or the *fr* limits by inserting Eqs (10) into (20) and performing the integral in *ξ* numerically.

## Numerical Computations

We compute the theoretical expressions of the relative errors that we obtain with our theory and compare them with the asymptotic expressions presented in [1, 6, 7] using the parameters listed in Table 1 and *D*_*S*_ = 0.

**Table 1:** Parameters used to make the curves of Fig 1. These parameters were chosen arbitrarily.

	Fig 1(a) and 1(b)	Fig 1(c)	Fig 1(d)
*D*_*f*_	1*μm*^2^ *s*^−1^	10*μm*^2^ *s*^−1^	1*μm*^2^ *s*^−1^
*k*_*off*_	10*s*^−1^	0.1*s*^−1^	100*s*^−1^
*K*_*D*_	1.66*μM*	16.6*nM*	0.083*μM*
[*P*^(*f*)^]	0.33*μM*	83.1*nM*	16.6*μM*
[*S*_*T*_]	3.32*μM*	16.6*nM*	11.63*μM*
*V*_*obs*_	0.125*μm*^3^	0.125*μm*^3^	1*μm*^3^

We perform stochastic numerical simulations of the reaction-diffusion system considered as in [16]. To compute *N*^(*s*)^(*t*) we count all the particles inside a cube of size 0.016*μm*^3^ located at the center of the integration volume. We use *D*_*S*_ = 0 and the parameters listed in Table 2. The diffusion coefficients and the ratios between the dissociation constant and the various concentrations of the first column of the Table are the same as those derived from an analysis [10] of FCS experiments performed in *Drosophila melanogaster* embryos. These parameters are such that the fast reaction limit holds. The concentrations and the dissociation constant of the second column were chosen so that the fast diffusion limit was satisfied. For the simulations the corresponding ACFs are computed as $G^{\left(s\right)}\left(\tau =jdt\right)=\sum_{\ell =0}^{n-1}(N^{\left(s\right)}\left(\ell dt\right)-\langle N^{\left(s\right)}\rangle )(N^{\left(s\right)}\left(\left(\ell +j\right)dt\right)-\langle N^{\left(s\right)}\rangle )/n$ with *dt* the time step. We generate the data points performing a very long simulation and subsenquently dividing the data into records of length, *T*_*obs*_ = 100*s*.

**Table 2:** Parameters used in the stochastic simulations. The parameters of the first column were derived in [10] from an analysis of FCS experiments performed in *Drosophila* embryos. Those of the second column were chosen arbitrarily.

	Fig 2(a)	Fig 2(b)
*D*_*f*_	19*μm*^2^ *s*^−1^	19*μm*^2^ *s*^−1^
*k*_*off*_	400*s*^−1^	0.5*s*^−1^
*K*_*D*_	0.25*μM*	1.92*nM*
[*S*]	2.87*μM*	22.1*nM*
[*P*^(*f*)^]	7.68*μM*	59.1*nM*
[*P*^(*b*)^]	88.31*μM*	679.3*nM*

## Results

In this paper we consider a system of free particles, *P*^(*f*)^, that diffuse and react with (immobile) binding sites, *S*, giving rise to bound particles, *P*^(*b*)^ (see Eq 8), as an idealization of several situations that are encountered in biological systems. In one of the two examples that are described later in more detail *P*^(*f*)^ represents transcription factors and *S* regulatory sites for the expression of different genes. In the other example, *P*^(*f*)^ is a substrate and *S* the enzyme that transforms it into a product. Given that the processes of interest involve the interaction of individual molecules to individual binding sites that occurs when the two “actors” are sufficiently close together the problem that we are dealing with is to what extent the rate at which these individual reactions occur is a good indicator of the particles concentration in the bulk, *i.e.* beyond the rather limited interaction (or “observation”) volume, *V*_*o*_. Even if the binding sites are immobile (as in the examples considered in the present paper), free particles keep on arriving in their vicinity and the reactions keep on occurring along time. Thus, there is an averaging process by which the rate eventually becomes a good indicator of the bulk concentration. The question then reduces to the time it takes for this to happen. For the problem at hand we idealize the problem by considering the time it takes for the averages, ${\bar{N}}^{\left(f\right)}$ and ${\bar{N}}^{\left(b\right)}$, of the number of free, *N*^(*f*)^, and bound, *N*^(*b*)^, particles in *V*_*o*_, respectively, to be within a certain percent of the corresponding means, 〈*N*^(*f*)^〉 and 〈*N*^(*b*)^〉, which are functions of the bulk concentration, [*P*^(*f*)^]. In S1 Text we describe the way by which we compute the relative errors, $\Delta_{r}\left({\bar{N}}^{\left(f\right)}\right)$ and $\Delta_{r}\left({\bar{N}}^{\left(b\right)}\right)$ of ${\bar{N}}^{\left(f\right)}$ and ${\bar{N}}^{\left(b\right)}$ and in Materials and Methods we present the main formulas that we obtain. In particular, building upon our previous work on the analysis of FCS experiments [10, 15–17] we derive analytic expressions for these errors that are valid for all times under different assumptions. In this Section we present a series of results that validate these analytic expressions and study the differences between our expressions and those previously published in the literature [1, 7] that only hold for long enough times. The calculations of S1 Text involve the linearization of a nonlinear problem. In this Section we also present an extension of our formulas that goes a step further into the nonlinear regime which is relevant for the early decay of the errors. We also derive an approximation of the dwell time distribution between individual bindings that we can obtain because we have expressions that are valid for early times. The applications of the following Section highlight the relevance of having analytic expressions that are valid beyond the asymptotic (long time) regime.

### Relative errors: early and asymptotic decay

**Fig 1:**
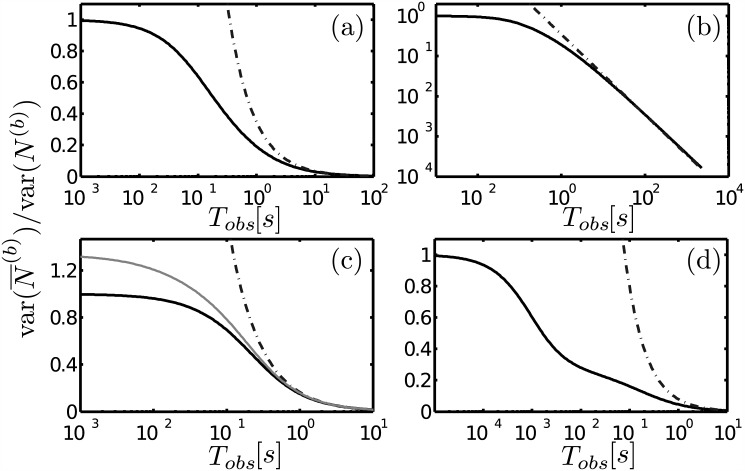
Relative error of the average number of bound particles as a function of time. Eq (7) with *τ*^(*b*)^ given by Eq (12) (dashed-dotted curve) is plotted in all subfigures. (a) The *fr* limit holds. Eq (24) (black solid curve) and Eq (25) (dashed-dotted curve) are also plotted. (b) Same as (a) but with the vertical axis on a logarithmic scale. (c) The *fd* limit holds. Eq (22) (black solid curve) and the combination of Eq (3) and Eq (27) (gray solid curve) are also plotted. (d) Similar to (a) but for parameters such that $\Delta_{r}\left({\bar{N}}^{\left(b\right)}\right)$ goes below 10% for *T*_*obs*_ much smaller than the longest correlation time which is ∼1*s*.

We here compare the time dependence of the relative errors that we derive with our theory with the asymptotic expressions presented in [1, 6, 7]. We show in Fig 1 the error, $\Delta_{r}\left({\bar{N}}^{\left(b\right)}\right)$, as a function of the observation time, *T*_*obs*_, given by Eq (22) (c) or Eq (24) (a,b,d) (black solid curves) and the one given by Eq (7) with *τ*^(*b*)^ given by Eq (12) (dashed-dotted curves). We use *D*_*S*_ = 0 and the parameters listed in Table 1. These parametes were chosen arbitrarily to highlight the main aspects that we want to stress about the difference between our formulas and the asymptotic ones. The *fd* limit holds in Fig 1(c) and the *fr* limit in Fig 1(a), 1(b) and 1(d) for which we used *β* = *p*_*b*_(1 − *p*_*b*_)/〈*N*^(*f*)^〉. We observe that the analytic approximations and the asymptotic expressions eventually predict the same decay. This is clearest in Fig 1(b). To illustrate the role of the two times, ${\tilde{\tau }}_{r}$ and *τ*_*ef*_, that characterize the decay in the *fr* limit we also plot in Fig 1(a), (b) and (d) the approximation given by Eq (25) (dashed curves). If ${\tilde{\tau }}_{r}⪡\tau_{ef}$, as in these examples, the term proportional to ${\tilde{\tau }}_{r}/T_{obs}$ in Eq (25) can be negligible already for times, *T*_*obs*_ ≪ *τ*_*ef*_. Furthermore, depending on the weights with which *τ*_*r*_ and *τ*_*ef*_ enter the expression of the error (1/(1 + *β*) and *β*/(1 + *β*), respectively), *Δ*_*r*_(*N*^(*b*)^) may drop to a very small value, *α*, for *T*_*obs*_ ≪ *τ*_*ef*_. In such a case, the asymptotic expression overestimates the time that is necessary for the error to drop below *α*. An extreme example of this situation (with *β* = 0.0034, *τ*_*r*_ = 50*μs* and *τ*_*ef*_ ≈ 1*s*) is shown in Fig 1(d). In this case *Δ*_*r*_(*N*^(*b*)^)∼0.1 for *T*_*obs*_ ≈ 1*ms* ≪ *τ*_*ef*_ while the asymptotic expression predicts that this error is reached at *T*_*obs*_ ≈ 100*ms*.

### Relative errors: comparison with stochastic simulations

**Fig 2:**
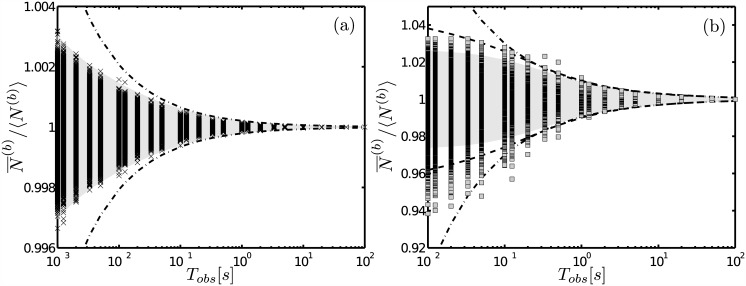
Comparing the predictions of the analytic expresions with stochastic simulations. We show the time-dependence of the normalized average numbers of bound particles, *N*^(*b*)^, obtained from stochastic simulations (symbols) and of the region defined by $1\pm 2\Delta_{r}\left({\bar{N}}^{\left(b\right)}\right)$ with $\Delta_{r}\left({\bar{N}}^{\left(b\right)}\right)$ computed in various ways. The dashed-dotted curves correspond to Eq (7) with *τ*^(*b*)^ given by Eq (12). (a) The *fr* limit holds. The shaded area is computed using Eq (24). (b) The *fd* limit holds. Eq (22) is used to compute the shaded area. The error derived from Eq (27) is also shown (dashed curve).

We now compare the predictions of the analytic expressions with stochastic simulations in which there are several binding sites inside the observation volume. The simulations are performed using the parameters of Table 2. We show in Fig 2 the (normalized) average number of bound particles in *V*_*obs*_ obtained from the simulations (symbols) as a function of *T*_*obs*_. We also plot the curves 1±2*Δ*_*r*_(*N*^(*b*)^) and shade the region between them for different expressions of *Δ*_*r*_(*N*^(*b*)^): Eq (7) (dashed-dotted curve), Eq (24) (shaded region in (a)) and Eq (24) (shaded region in (b)). In Fig 2(a) the *fr* limit holds so that *Δ*_*r*_(*N*^(*b*)^) (see *e.g.* Eq (24) depends on *β*, *i.e.*, on the linearization that is used. We have used the linearization that leads to Eq (12) [1] and obtained *β* = *p*_*b*_(1 − *p*_*b*_)〈*N*_*ST*_〉/〈*N*^(*f*)^〉 = 0.36 for the parameters of the simulation. We observe that the theoretical expression describes correctly the decay of the relative errors obtained with the simulations. In Fig 2(b) the *fd* limit holds. In this limit, both linearizations lead to the same ACFs (Eqs (14) and (15)). We observe that Eq (22) (the one that defines the shaded region) underestimates the size of the fluctuations at early times. We discuss in what follows a possible reason for this to happen and introduce the correction that is illustrated with the dashed curve.

### Initial error: including the effect of nonlinearities

For immobile binding sites *N*^(*b*)^ is binomial and var(*N*^(*b*)^) = (1 − *p*_*b*_)〈*N*^(*b*)^〉 depends on 〈*N*^(*f*)^〉 because *p*_*b*_ = 〈*N*^(*f*)^〉/(〈*N*^(*f*)^〉+*K*_*D*_ *V*_*obs*_). All the error estimates that we have used so far were derived assuming that the system was close to equilibrium and the dynamics could be modeled by a set of linear equations. The dependence of var(*N*^(*b*)^) on 〈*N*^(*f*)^〉 is a consequence of the nonlinearity of the problem. The number of free particles in *V*_*obs*_, *N*^(*f*)^, is a stochastic variable and it is subject to the same type of uncertainties as *N*^(*b*)^. Namely, it takes some time for ${\bar{N}}^{\left(f\right)}$ to differ from 〈*N*^(*f*)^〉 within a small percent. We may then consider that, after a time, *T*_*obs*_, there is only an approximation to *p*_*b*_ given by ${\tilde{p}}_{b}={\bar{N}}^{\left(f\right)}\left(T_{obs}\right)/\left({\bar{N}}^{\left(f\right)}\left(T_{obs}\right)+K_{D}V_{obs}\right))\langle N^{\left(b\right)}\rangle$ and that fluctuations in *N*^(*f*)^, which decay with their own correlation times, impact directly on var(*N*^(*b*)^) (and on $\mathrm{var}({\bar{N}}^{\left(b\right)})$). In order to take this effect into account we estimate $|{\tilde{p}}_{b}-p_{b}|$ as:

$$\Delta p_{b}=\left|\frac{\partial {\tilde{p}}_{b}}{\partial {\bar{N}}^{\left(f\right)}}\right|\sqrt{\mathrm{var}({\bar{N}}^{\left(f\right)})}=p_{b}\left(1-p_{b}\right)\Delta_{r}\left(\bar{N_{f}}\right),$$ (26)

then approximate:

$$\mathrm{var}\left(N^{\left(b\right)}\right)\approx \left(1-p_{b}\right)\langle N^{\left(b\right)}\rangle +\left|\frac{\partial \mathrm{var}(N^{\left(b\right)})}{\partial p_{b}}\right|\Delta p_{b}=\left(1-p_{b}\right)\langle N^{\left(b\right)}\rangle \left(1+p_{b}\Delta_{r}\left({\bar{N}}^{\left(f\right)}\right)\right),$$ (27)

and use it to compute $\Delta_{r}\left({\bar{N}}^{\left(b\right)}\right)$ as before. The “nonlinear” correction of Eq (27) should be noticeable if 〈*N*^(*f*)^〉 is small enough, a situation that is encountered when the *fd* limit holds. We show with dashed lines in Fig 1(c) the curve $\Delta_{r}\left({\bar{N}}^{\left(b\right)}\right)$ *vs* *T*_*obs*_ obtained as just described using Eq (21) to compute $\Delta_{r}\left({\bar{N}}^{\left(f\right)}\right)$. We show in Fig 2(b) with dashed-dotted lines the curves, $1\pm 2\Delta_{r}\left({\bar{N}}^{\left(b\right)}\right)$, obtained in a similar way. These curves decay with the additional correlation time, *τ*_*f*_ = *a*^2^/*D*_*f*_ = 7*ms*, with respect to those depicted with solid lines.

### Dwell-time distributions

Using the analytic expressions of the ACF we can derive an approximation of the distribution of times between successive bindings where there is a single binding site. To this end we recall that, if *N*_*ST*_ = 1, the ACF Eq (9) for *s* = *S* or *s* = *b* (the unbound and the bound binding site, respectively) can be interpreted as:

$$\begin{array}{cc} G^{\left(s\right)} & \left(\tau \right)=G^{\left(b\right)}\left(\tau \right)\\ & =\left(1-p_{b}\right)\left(P\left(\phi_{S},t+\tau |\phi_{S},t\right)-\left(1-p_{b}\right)\right)\\ & =\left(1-p_{b}\right)\left(1-P\left(\phi_{b},t+\tau |\phi_{S},t\right)-\left(1-p_{b}\right)\right) \end{array}$$ (28)

with *p*_*b*_, as before, the equilibrium probability that the site be bound and $P(\phi_{s},t\;+\;\tau |\phi_{s}^{\prime },t)$ the probability that the site be in state *s* at time *t*+*τ* given that it was in state *s*′ (*s*, *s*′ = *S*, *b*) at time, *t*. We must note that the site may switch state (*S*, *b*) several times between *t* and *t*+*τ*. However, if *τ* is not too large (it is of the order of or smaller than the reaction time, *τ*_*r*_), we may assume that at most one switching occurs. Let us assume that at time, *t*, a transition from bound (*b*) to free (*S*) occurs. So, at time, *t*, the site is free. Then, if *τ* is not too large,

$$f\left(\tau \right)\approx \zeta \frac{\partial }{\partial \tau }P\left(\phi_{b},t+\tau |\phi_{S},t\right)=-\zeta \frac{dG^{\left(b\right)}}{d\tau }/\left(1-p_{b}\right),$$ (29)

with *ζ* a normalization factor, gives an estimate of the transition probability per unit time for the site to become bound, *i.e.* of the distribution of waiting times between bindings. The normalization factor *ζ* is chosen so that $\int_{0}^{\infty }d\tau f\left(\tau \right)=1$. Using the analytic expressions of the ACF and Eq (29) we derive the following approximations of the dwell-time distribution in the *fd* and the *fr* limits:

$$f_{fd}\left(\tau \right)=\frac{1}{\tau_{off}}e^{-\tau /\tau_{off}},$$ (30)

$$f_{fr}\left(\tau \right)=\zeta \left(\frac{3}{2\tau_{ef}}\frac{\beta }{{\left(1+\frac{\tau }{\tau_{ef}}\right)}^{5/2}}+\frac{1}{\tau_{r}}e^{-\tau /\tau_{r}}\right).$$ (31)

The distribution is exponential in the former and has a long tail in the latter. As we discuss later this different behavior can explain the transition in the dwell-time distribution observed [18] in assays of enzyme activity at the single molecule level.

## Discussion

Information transmission in cells occurs quite accurately even when concentration changes are “read” by individual target molecules. In this paper we have studied molecule number fluctuations as a way to answer the question of how long it takes for a concentration to be “read” by binding sites with a certain accuracy. As done in [1, 6, 7], we have approximated the error of the concentration estimate in a region in terms of the variance of the average number of molecules in that region after an observation time, *T*_*obs*_. Following [6] we have used the ACF of the molecule number fluctuations, *G*^(*s*)^(*τ*), to compute this variance. Thus, it is the characteristic (correlation) times of the ACF, *τ*_*i*_, the ones that determine how fast the concentration can be estimated. There are two main differences of our approach with respect to previous works [1, 6, 7]. One one hand, we have analyzed the case in which the molecules whose concentration is to be read do not interact with a single binding site. On the other hand, we have obtained expressions for the variance and, thus, for the relative error, Eq (3), that hold for all times, not only for *T*_*obs*_ ≫ max_*i*_ *τ*_*i*_. Our expressions described very well the decay of the fluctuations obtained in particle simulations as illustrated in Fig 2. The limitation of our expressions is that they are approximations that hold in two opposite limits, the *fast diffusion* and the *fast reaction* one. The former limit holds when the characteristic diffusion and reaction times defined in Eq (13) satisfy *τ*_*f*_ ≪ *τ*_*r*_. The latter holds when *τ*_*r*_ ≪ *τ*_*f*_. The *fd* limit becomes valid, in general, as the observation volume is reduced [16]. The *fr* approximation of the auto-correlation functions, *G*^(*s*)^(*τ*), is always valid if the lag time, *τ*, is sufficiently large [17]. The transition between both limits was studied in [16]. There it was observed that even for *τ*_*r*_ ∼ *τ*_*f*_ the *fr* approximation provided a good description of the ACFs. Even though there are real situations that do not fit within any of the two limits, knowing the behavior of the ACFs for both of them gives an indication of what may happen in between [16]. In these limits the ACFs are approximately of the form:

$$G^{\left(s\right)}=\mathrm{var}(N^{\left(s\right)})\sum_{i}W_{i}^{\left(s\right)}\Phi_{i}^{\left(s\right)}\left(\tau \right)$$ (32)

with $\Phi_{i}^{\left(s\right)}\left(0\right)=1$, $\sum_{i}W_{i}^{\left(s\right)}=1$ and $\Phi_{i}^{\left(s\right)}\left(\tau \right)$ characterized by a single correlation time, *τ*_*i*_. Thus, the asymptotic correlation time, Eq (6), studied in [1, 6, 7], is a weighted average of the individual correlation times, *τ*_*i*_. The relative errors, Eqs (21)–(25), on the other hand, satisfy:

$$\Delta_{r}\left({\bar{N}}^{\left(s\right)}\right)\approx \sqrt{\mathrm{var}(N^{\left(s\right)})}/\langle N^{\left(s\right)}\rangle \;\text{if}\;T_{obs}\ll \min_{i}\tau_{i},$$ (33)

while they approach the asymptotic expression (the equivalent of Eq (7) for each species, *s*) for *T*_*obs*_ ≫ max_*i*_ *τ*_*i*_. We have shown in Fig 1 how, depending on the weights, using the asymptotic expression (7) can lead to an overestimation of the time it takes for $\Delta_{r}\left({\bar{N}}^{\left(b\right)}\right)$ to go below a certain value. This highlights the relevance of having a formula that describes the fluctuations decay before the asymptotic behavior is valid. Eq (33) implies that, at early times, $\Delta_{r}\left({\bar{N}}^{\left(b\right)}\right)\approx \left(1-p_{b}\right)\langle N^{\left(b\right)}\rangle$, which depends on 〈*N*^(*f*)^〉 through *p*_*b*_. *N*^(*f*)^ is a stochastic variable on equal grounds as *N*^(*b*)^ and it takes a while for its average, ${\bar{N}}^{\left(f\right)}$, to reach its expected value, 〈*N*^(*f*)^〉. In this paper we have extended the expression for $\Delta_{r}\left({\bar{N}}^{\left(b\right)}\right)$ to take this nonlinear effect into account. This extended expression improved the description of the early decay of the error in the *fd* limit, as illustrated in Fig 2(b). As discussed later, this extended expression allows to explain the co-existence observed in *Drosophila* embryos [4] of highly fluctuating instantaneous transcriptional activities and of accumulated mRNA concentrations with noise levels that are reduced beyond what time-averaging predicts. Having an analytic expression of the ACF for all times also allowed us to derive approximations for the distribution of waiting times between successive bindings. They are given by Eqs (30) and (31) in the *fd* and the *fr* limits, respectively. The different time-dependence of both expressions, which is a consequence of whether diffusion or reactions are the time-limiting steps, is lost when one looks at the asymptotic correlation time Eq (6). As discussed later, it can be used to explain the changes in enzyme activity observed as the substrate concentration is increased [18] in a way that does not rely on the existence of innumerous enzyme conformers.

### The collective diffusion coefficient determines the correlation time of bound binding site number fluctuations when there are several binding sites in the observation volume

The problem of how long it takes for a single binding site to “read” the concentration of its ligand has recently been revisited in [7]. In particular the authors analyzed the different dependence of the asymptotic time, *τ*^(*b*)^, obtained in [6] and in [1]. This difference is apparent in the first term of Eqs (11) and (12) which differ in the factor (1 − *p*_*b*_) that is present in the latter. This first term dominates *τ*^(*b*)^ in the *fr* limit. Thus, it is in this limit that the different predictions could be distinguished provided that (1 − *p*_*b*_) is sufficiently different from 1. The example probed in Fig 2(a) satisfies this condition (1 − *p*_*b*_ = 0.03). The theoretical expressions that were used in Fig 2(a) correspond to the linearization that leads to the asymptotic time, Eq (12), derived in [1]. In particular, we used Eq (24) with *β* = *p*_*b*_(1 − *p*_*b*_)*N*_*ST*_/〈*N*^(*f*)^〉 = 0.36 to determine the shaded area of Fig 2(a). We observe that these theoretical expressions describe correctly the size of the early fluctuations and the way they decay asymptotically in time. Had we used the other linearization (the one that leads to Eq (11) [7]) the value *β* = *p*_*b*_ *N*_*ST*_/〈*N*^(*f*)^〉 = 11.5 would have been used in Eq (24) instead. In such a case, the theoretical expressions of the relative error would have been ∼32 times larger than those depicted in Fig 2(a) and would not describe the observed fluctuations so well. In this example the volume that is probed contains many (∼20,000) binding sites. When there are several (independent) binding sites on a surface the calculation of [14] shows that the asymptotic time approaches the one given by Eq (12). Our simulation supports this result. It is interesting to note that one of the differences between the results that are obtained using the one or the other linearizations described in S1 Text is the different correlation time that dominates the asymptotic decay of the fluctuations in the *fr* limit. In both cases it is a diffusive time but with the two different effective diffusion coefficients introduced in [11]: the collective one in the case that the asymptotic time is given by Eq (12) and the single molecule one that of Eq (11). The former describes the time it takes for a perturbation in the concentration of particles to spread out while the latter prescribes how the mean square displacement of a single particle scales with time [11]. The result about the correlations in the occupancy state of a single binding site (the case probed in [7]) as compared to a collection of them (the case probed here) indicates that a similar difference between “single” and “collective” behavior also occurs for the binding sites. The transition between both situations will be studied in the future.

### The free diffusion coefficient of Bicoid sets the limit with which its concentration can be read

In [3] the time, *T*_*obs*_, it takes for the concentration of the transcription factor, Bcd, to be known with a 10% precision in the region of the embryo that shows an abrupt change in the concentration of the protein whose production it regulates, Hb, was estimated using *Δ*_*r*_[*Bcd*]∼(*aD*[*Bcd*]*T*_*obs*_)^−1/2^ with *D* ∼ 1*μm*^2^/*s*, the Bcd diffusion coefficient obtained with FRAP [8], *a* ≈ 3*nm*, the typical size of a DNA binding site and [*Bcd*]∼5/*μm*^3^. The authors argued that this expression gave a lower bound of *T*_*obs*_ (the term inversely proportional to *k*_*off*_ in Eq (12) was not considered). However, the value obtained, *T*_*obs*_ ∼ 7000*s* ≈ 2*h*, was too long. Identifying Bcd with the species, *P*^(*f*)^, of our model we can estimate *T*_*obs*_ in two ways. One, by considering the time it takes for $\Delta_{r}\left({\bar{N}}^{\left(f\right)}\right)\approx 0.1$ in the volume, *V*_*obs*_ ∼ *a*^3^, probed by a binding site. *V*_*obs*_ is very small. Thus, we may use Eq (21) with var(*N*^(*f*)^) = 〈*N*^(*f*)^〉 to compute $\Delta_{r}\left({\bar{N}}^{\left(f\right)}\right)$ which corresponds to the “perfect measuring” device of [6]. For *T*_*obs*_ ≫ *τ*_*f*_ this leads to:

$$\Delta_{r}\left({\bar{N}}^{\left(f\right)}\right)={\left(\frac{4a^{2}}{\langle N^{\left(f\right)}\rangle D_{f}T_{obs}}\right)}^{1/2},$$ (34)

which coincides with the expression used in [3] if we set *V*_*obs*_ = 4*a*^3^ and *D* = *D*_*f*_, the free diffusion coefficient of Bcd. According to the analysis of [10], the latter is ∼20 times larger than the effective coefficient that can be estimated with FRAP which was used in [3]. Thus, using the free diffusion coefficient, *D*_*f*_, the resulting *T*_*obs*_ ∼ 350*s* ∼ 6*min* is 20 times smaller than the one derived in [3]. It is worth noticing that Eq (34) is also obtained if we use the *fr* limit expression, Eq (23), to compute $\Delta_{r}\left({\bar{N}}^{\left(f\right)}\right)$. The second way of computing *T*_*obs*_ is by considering the time it takes for $\Delta_{r}\left({\bar{N}}^{\left(b\right)}\right)$ to be such that

$$\Delta_{r}\left({\bar{N}}^{\left(f\right)}\right)\approx \Delta_{r}\left({\bar{N}}^{\left(b\right)}\right)\frac{\langle N^{\left(b\right)}\rangle }{\langle N^{\left(f\right)}\rangle }/\left|\frac{d(\langle N^{\left(b\right)}\rangle )}{d(\langle N^{\left(f\right)}\rangle )}\right|\approx 0.1,$$ (35)

with 〈*N*^(*b*)^〉 = *p*_*b*_ = 〈*N*^(*f*)^〉/(〈*N*^(*f*)^〉+*K*_*D*_ *V*_*obs*_). This is the approach followed in [1, 6, 7]. Although *V*_*obs*_ is very small, we may argue that, since there is one fixed binding site in it, the corresponding concentration, [*S*_*T*_] = 1/*V*_*obs*_ so that Eq (24) with var(*N*^(*b*)^) = (1 − *p*_*b*_)〈*N*^(*b*)^〉 should be used. In the limit of large enough *T*_*obs*_, this leads to:

$${\left(\Delta_{r}\left({\bar{N}}^{\left(b\right)}\right)\right)}^{2}=\frac{1-p_{b}}{\langle N^{\left(b\right)}\rangle }\frac{4a^{2}\beta }{D_{f}T_{obs}}.$$ (36)

Combining Eqs (36) and (35) we obtain

$$\Delta_{r}\left({\bar{N}}^{\left(f\right)}\right)\approx {\left(\frac{4a^{2}\beta }{p_{b}\left(1-p_{b}\right)D_{f}T_{obs}}\right)}^{1/2}$$ (37)

which, for *β* = *p*_*b*_/〈*N*^(*f*)^〉, is similar to the formula used in [6, 7] and, for *β* = *p*_*b*_(1 − *p*_*b*_)/〈*N*^(*f*)^〉, to the one used in [1] if we again identify *D* = *D*_*f*_ and *V*_*obs*_ = 4*a*^3^. In particular, the latter leads to Eq (34), thus, to the same estimate for *T*_*obs*_ as before. The former leads to a similar expression for $\Delta_{r}\left({\bar{N}}^{\left(f\right)}\right)$ but where the right hand side is additionally divided by 1 − *p*_*b*_. Assuming for simplicity that [*Hb*]∝*p*_*b*_ = 〈*N*^(*f*)^〉/(〈*N*^(*f*)^〉 + *K*_*D*_ *V*_*obs*_) we estimate that the sharp transition in [Hb] occurs where 〈*N*^(*f*)^〉≈*K*_*D*_ *V*_*obs*_, *i.e.*, where *p*_*b*_ ≈ 1/2. Using *a* = 3*nm*, [*P*^(*f*)^]∼5/*μm*^3^ and *D*_*f*_ = 20*μm*^2^/*s* we obtain *T*_*obs*_ ∼ 700*s* ∼ 12*min* for $\Delta_{r}\left({\bar{N}}^{\left(f\right)}\right)∼0.1$. The use of the free diffusion coefficient estimate of [10] is fundamental to having derived these estimates of *T*_*obs*_ that are smaller than the time it takes for the gradient of Bcd to get established. It is important to note, however, that all these conclusions hold provided that the regulatory sites on the DNA are the only ones to/from which Bcd binds/unbinds. If Bcd also interacts with other sites (*e.g.*, mRNA) and it is these other interactions that introduce the main limitations to the transport of Bcd, we expect the resulting collective diffusion coefficient to be the one that determines the arrival times of free molecules and the precision with which their concentration can be “read” by the regulatory sites. This needs, however, a separate study that will be done in the future.

### The “early” spatial averaging of transcription factor concentrations can explain the large noise reduction observed in *Drosophila* in the resulting accumulated mRNA

The work of [4] addressed the problem of how the accuracy of the reading of Bcd is achieved by a direct observation of the dynamics of transcription. To this end they used a construction which fluorescence reports the nascent mRNA content inside a 4.5*μm*^3^ volume. The experiments showed a ∼45% fluctuation level at each transcription locus which was compared with that of the accumulated mRNA. The experiments were done for Hb and for the gene, Krüppel (kr), because the accumulated mRNA of the latter is proportional to the elapsed time. To analyze the observations the authors argued that if transcription has standard deviation, *σ*_*nuc*_, per *N*_0_ mRNA molecules produced, the maximum noise reduction in the accumulated mRNA is achieved by independently running the process *m* times. Taking into account the measurement noise, *η*, the fractional noise, ${\hat{\sigma }}_{cyto}$ (what we call the relative error), of the total (accumulated) mRNA produced up to a certain time and that of the instantaneous transcription, ${\hat{\sigma }}_{nuc}=\sigma_{nuc}/N_{0}$, are related by:

$${\hat{\sigma }}_{cyto,L}={\left({\hat{\sigma }}_{nuc}^{2}N_{0}/\mu +\eta^{2}\right)}^{1/2},$$ (38)

where *μ* is the mean of the accumulated mRNA and where the subscript *L* is used to distinguish this expression from the one we derive later that takes into account a “nonlinear” correction to estimate the error. From the observations the authors obtain *N*_0_ = 100±20 and ${\hat{\sigma }}_{nuc}=0.22\pm 0.03$ and estimate *η* ≈ 0.03. According to this theory, by the time the mean expression level reaches 800 molecules per nucleus, ${\hat{\sigma }}_{cyto}\geq 0.08$. The observations of the accumulated mRNA, however, showed lower noise levels of 6%±2%. Thus, the authors concluded that time averaging could not account by itself for the reduction in fluctuations observed when going from instantaneous transcription to accumulated mRNA.

**Fig 3:**
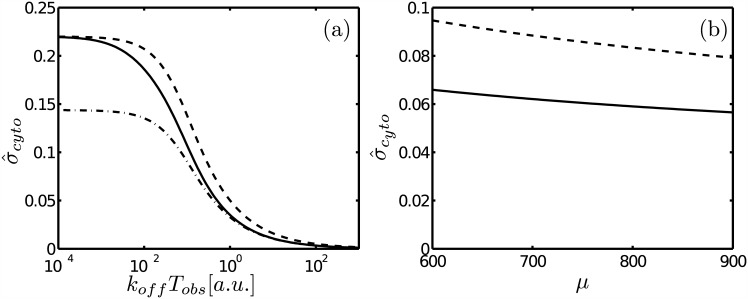
Comparison of different theoretical prescriptions of the fractional noise, ${\hat{\sigma }}_{cyt}$, as time increases. (a) ${\hat{\sigma }}_{cyt}$ *vs* *T*_*obs*_ computed using Eq (22) with ${\hat{\sigma }}_{cyt}\left(T_{obs}=0\right)=0.22$ (dashed line) and with ${\hat{\sigma }}_{cyt}\left(T_{obs}=0\right)=0.22/\left(1+p_{b}/\sqrt{\langle N^{\left(f\right)}\rangle }\right)$ (dashed-dotted lines) and using the combination of Eqs (3) and (27) with ${\hat{\sigma }}_{cyt}\left(T_{obs}=0\right)=0.22$ (solid line). (b) Two of the curves displayed in (a) using the same symbols as in (a) but as functions of the mean of the accumulated mRNA produced. The curves correspond to Eq (38) (dashed) and Eq (42) (solid) with ${\hat{\sigma }}_{nuc}=0.22$ in both cases.

Although our model is simple it gives some insight into the processes that might be responsible for the observed noise reduction. To this end we assume that fluctuations in the instantaneous nascent mRNA content are proportional to fluctuations in *N*^(*b*)^ and that those in the mRNA accumulated up to *T* are proportional to fluctuations in $\int_{0}^{T}dtN^{\left(b\right)}=T{\bar{N}}^{\left(b\right)}$. Thus, the fractional noises, ${\hat{\sigma }}_{nuc}$ and ${\hat{\sigma }}_{cyto}$ (after a time, *T*_*obs*_) are given, respectively, by $\Delta_{r}\left({\bar{N}}^{\left(b\right)}\right)\left(T=0\right)$ and $\Delta_{r}\left({\bar{N}}^{\left(b\right)}\right)\left(T=T_{obs}\right)$. The expressions of $\Delta_{r}\left({\bar{N}}^{\left(b\right)}\right)\left(T_{obs}\right)$ given by Eqs (20), (22) or (24) decay as 1/*T*_*obs*_ for long enough time, which is the type of reduction produced by time averaging. The early decay of Eqs (22) and (24) is slower than 1/*T*_*obs*_ (see Fig 1). The extended expression that takes into account the time it takes for the value, *p*_*b*_, that is “sensed” by the binding site to converge to its expected value (Eq (26)) decays faster for short times (see Fig 1(c)). This allows us to explain the observed reduction in the size of the fluctuations as we discuss now. Eqs (27) and (33) imply that, for *T* ≈ 0, it is

$${\hat{\sigma }}_{nuc}^{2}=\frac{\mathrm{var}(N^{\left(b\right)})}{{\langle N^{\left(b\right)}\rangle }^{2}}\approx {\hat{\sigma }}_{nuc,L}^{2}\left(1+p_{b}\frac{\sqrt{\mathrm{var}(N^{\left(f\right)})}}{\langle N^{\left(f\right)}\rangle }\right),$$ (39)

where ${\hat{\sigma }}_{nuc,L}=\sqrt{\left(1-p_{b}\right)\langle N^{\left(b\right)}\rangle }/\langle N^{\left(b\right)}\rangle$ would have been the fractional noise of the instantaneous transcription had we not taken into account the “error” of *p*_*b*_. The general expression of the fractional noise, ${\hat{\sigma }}_{cyto}$, as a function of *T*_*obs*_ is a little lengthy. However, to make our point it suffices to show how it reads for *T*_*obs*_ large enough. To this end we replace $\Delta_{r}\left({\bar{N}}^{\left(f\right)}\right)$ in Eq (27) by its asymptotic value, Eq (34), and insert the result into Eq (7). We obtain:

$${\hat{\sigma }}_{cyto,NL}^{2}={\left(\Delta_{r}\left({\bar{N}}^{\left(b\right)}\right)\right)}^{2}\approx \frac{2\tau^{\left(b\right)}}{T_{obs}}{\hat{\sigma }}_{nuc,L}^{2}\left(1+p_{b}{\left(\frac{4a^{2}}{\langle N^{\left(f\right)}\rangle D_{f}T_{obs}}\right)}^{1/2}\right),$$ (40)

where we have used the subscript *NL* to denote that we are using the extended (nonlinear) expression for $\Delta_{r}\left({\bar{N}}^{\left(b\right)}\right)$. Eq (40) implies that $\Delta_{r}\left({\bar{N}}^{\left(b\right)}\right)$ is eventually given by Eq (7) with var(*N*^(*b*)^) = (1 − *p*_*b*_)〈*N*^(*b*)^〉 regardless of whether we use Eq (27) or var(*N*^(*b*)^) = (1 − *p*_*b*_)〈*N*^(*b*)^〉 for the (initial) variance of *N*^(*b*)^. The time, *τ*_*f*_ ∼ *a*^2^/*D*_*f*_, that characterizes the decay of the last term in the r.h.s of Eq (40) is the shortest correlation time of the problem for small enough *a*. Thus, although $\Delta_{r}\left({\bar{N}}^{\left(b\right)}\right)={\hat{\sigma }}_{cyto}$ is initially very large ($\Delta_{r}\left({\bar{N}}^{\left(b\right)}\right)\left(T=0\right)={\hat{\sigma }}_{nuc}$), part of it decays rapidly (with timescale, *τ*_*f*_). After this initial reduction the fractional noise is correctly described by Eq (22) in the *fd* or Eq (24) in the *fr* limits, *i.e.*, it decays as prescribed by time-averaging only, but with $\mathrm{var}\left(N^{\left(b\right)}\right)/{\langle N^{\left(b\right)}\rangle }^{2}={\hat{\sigma }}_{nuc,L}^{2}<{\hat{\sigma }}_{nuc}^{2}$. Including measurement errors as in [4] it can be expressed as:

$${\hat{\sigma }}_{cyto,NL}\approx \sqrt{\frac{{\hat{\sigma }}_{nuc,L}^{2}}{m}+\eta^{2}},\;\text{for}\;T_{obs}\gg \tau_{f},$$ (41)

where we have identified the number of times that the (binding) process is repeated, *m*, with *T*_*obs*_/(2*τ*^(*b*)^). Inserting Eqs (39) into (41) we obtain

$${\hat{\sigma }}_{cyto,NL}\approx {\left(\frac{{\hat{\sigma }}_{nuc}^{2}N_{0}}{\mu (1+p_{b}/{\langle N^{\left(f\right)}\rangle }^{1/2})}+\eta^{2}\right)}^{1/2},$$ (42)

where we have replaced *m* = *μ*/*N*_0_ as in [4] and var(*N*^(*f*)^) = 〈*N*^(*f*)^〉 since *N*^(*f*)^ is Poisson distributed. Comparing Eqs (42) and (38) we see that the nonlinear correction explains a larger noise reduction than the one predicted by time-averaging. This additional reduction in the fractional noise can be associated to the spatial averaging of the transcription factor, *P*^(*f*)^, not of the product, as considered in [4]. We check now whether this reduction can be quantitatively similar to the one observed experimentally. In [4]${\hat{\sigma }}_{nuc}∼0.22$ was estimated observing the instantaneous transcription occurring in a few (at most four in the case of Bcd) active genomic loci in each nucleus that were indistinguishable due to experimental limitations. Given that Bcd binds cooperatively to modulate the production of Hb, it is likely that there could be more binding sites in the observation volume. Furthermore, all these details are unknown in the case of Kr. Thus, we use *N*_*ST*_ = 6 to quantify the reduction. The rest of the parameters correspond to the estimates obtained for Bcd in [10] (*D*_*f*_ = 19*μm*^2^/*s*, [*P*^(*f*)^] = 7*nM*, *K*_*d*_ = 0.192*nM*), to the ones derived in [4] (*N*_0_ = 100, *η* = 0.03, ${\hat{\sigma }}_{nuc,NL}=0.22$), were chosen so as to reproduce the observations (*V*_*obs*_ = 0.125*μm*^3^) or arbitrarily within reasonable values (*k*_*off*_ = 1/*s*). We show in Fig 3 plots of ${\hat{\sigma }}_{cyto}=\Delta_{r}\left({\bar{N}}^{\left(b\right)}\right)$ computed using the *fd* limit, Eq (22), with different expressions for var(*N*^(*b*)^)/〈*N*^(*b*)^〉^2^. The dashed and the dashed-dotted curves correspond to considering a fixed value, var(*N*^(*b*)^)/〈*N*^(*b*)^〉^2^ = (1 − *p*_*b*_)/〈*N*^(*b*)^〉, so that the reduction is limited by time averaging over the timescale, *τ*^(*b*)^. The solid curve corresponds to considering it is given by the time-dependent expression (27) with *Δ*_*r*_(*N*^(*f*)^) given by Eq (21), so that there is the additional initial reduction over the timescale, *τ*_*f*_. The dashed and solid curves start from the same initial value, ${\hat{\sigma }}_{cyto}\left(T=0\right)={\hat{\sigma }}_{nuc}=0.22$[4]. The dashed-dotted curve starts from a similar value to the one that $\Delta_{r}\left({\bar{N}}^{\left(b\right)}\right)$ reaches once “fluctuations in *p*_*b*_” have become negligible, ${\hat{\sigma }}_{cyto}\left(T_{obs}=0\right)∼{\hat{\sigma }}_{nuc,L}={\hat{\sigma }}_{nuc,NL}/\left(1+p_{b}/\sqrt{\langle N^{\left(f\right)}\rangle }\right)$. We observe in Fig 3(a) that the initial reduction of the fractional noise level over the timescale, *τ*_*f*_, gives similar results to having started with a smaller value of ${\hat{\sigma }}_{cyto}$. In other words, the fractional noise level of instantaneous transcription includes fluctuations in *N*^(*f*)^ that lead to an overestimation of (1 − *p*_*b*_)〈*N*^(*b*)^〉 if we assume that this expression represents the variance of the observed variable at these initial stages. We show in Fig 3(b) two of the curves displayed in Fig 3(a) (using the same symbols as in (a)) but as functions of the accumulated mRNA, *μ*. It is apparent in this figure that including the additional reduction of the initial fluctuations the experimental observations depicted in Fig S6 of [4] can be reproduced very well. Besides the quantitative agreement between our formulas computed using realistic parameter values and the observations of [4] which Fig 3 illustrates it is important to stress the different mechanism that is invoked to explain the observations in [4] and in the present paper. Given that the smoothing produced by time averaging is not enough, both mechanisms rely on some sort of spatial averaging. The authors of [4] suggest that the spatial mixing of the mRNA synthesized in different transcription sites can provide this spatial averaging. Our explanation argues that it is the variability in the number of transcription factors at the different sites that increase the fluctuations of the instantaneous transcription. As time goes by this variability decreases. Thus, in our explanation it is the spatial averaging of the transcription factors which is responsible for the early decay of the instantaneous transcription fluctuations that are subsequently smoothed out further by time averaging. The time-scales of both spatial averaging processes (the homogenization of the transcription factor or of the mRNA distributions) is different, so that these two explanations could be tested in experiments.

### The transition of the waiting time distribution observed in single-enzyme assays may be due to a change in the nature of the correlation times

**Fig 4:**
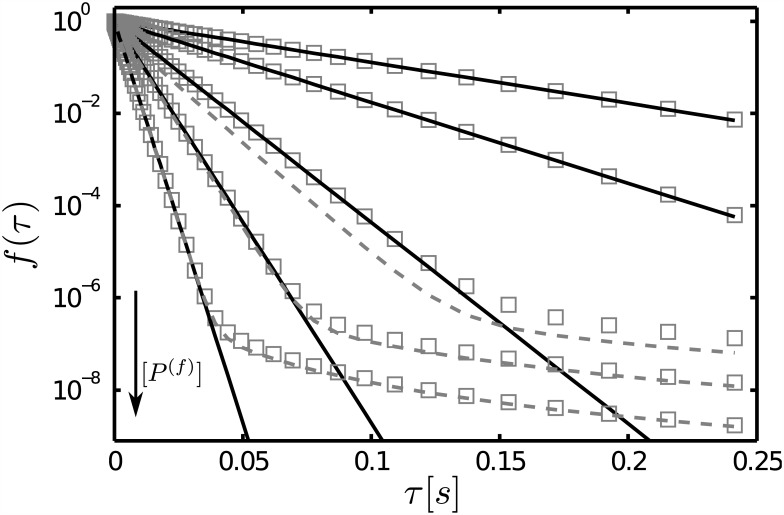
Normalized waiting time distribution between bindings of a single enzyme. We show with squares the dwell time distribution estimated differentiating Eq (10) as prescribed in Eq (29) and computing the integral numerically using *D*_*f*_ = 5*μm*^2^ *s*^−1^, *V*_*obs*_ = 5.6 × 10^−3^ *μm*^3^, [*S*_*T*_] = 1/*V*_*obs*_, *k*_*on*_ = 2*μM*^−1^ *s*^−1^, *k*_−1_ = 0.5*s*^−1^ and, from top to bottom, the enzyme concentrations [*P*^(*f*)^] = 10, 20, 50, 100 and 200*μM*. All the distributions are normalized with respect to their value at *τ* = 0. We also show the analytic expression (30) (solid curves) for all cases and Eq (31) (dashed curves) for the three cases with the largest values of [*P*^(*f*)^].

The scheme Eq (8) also corresponds to the first step of a Michaelis-Menten-like process in which a substrate, *P*^(*f*)^, binds to an enzyme, *S*, and is then transformed into a product at a rate proportional to [*P*^(*b*)^]. The dynamics of this type of process was observed at the single molecule level in [18]. The observations showed that the distribution of waiting times between individual turnovers was monoexponential at low [*P*^(*f*)^] and was characterized by several timescales at high [*P*^(*f*)^]. Although the experimental observations correspond to the generation of the product which involves (at least) an additional step with respect to Eq (8) (the one that goes from *P*^(*b*)^ to the product) increasing [*P*^(*f*)^] induces a similar change in the dwell-time distribution between successive bindings that we obtained in the Results Section. We show in Fig 4 with symbols a plot of the dwell-time distribution, *f*, obtained combining Eqs (29) and (10) and performing the integral numerically [16]. All parameters are fixed with the exception of [*P*^(*f*)^] that varies between curves. For comparison, we also show for certain cases the approximate expressions, Eqs (30) and (31), that hold, respectively, in the *fd* and *fr* limits. We observe that, in this example, one or the other approximation gives a good description of the distribution computed numerically. We also observe how *f* goes from being monoexponential to having a long tail with increasing [*P*^(*f*)^] as observed experimentally in [18]. Although a more accurate description would require the inclusion of the additional production step, we can describe this change as a transition from *f* being correctly described by the *fd* approximation to being described better by the *fr* one. While the first one is characterized by a single (reaction) time, the latter is characterized by a diffusive correlation time that is responsible for the long tail of the dwell-time distribution. Therefore, the transition can be associated to whether the reaction or the diffusive steps determine the dwell-time distribution without the need of having many different enzyme conformers.

## Supporting Information

S1 Text Model and calculations. In this text we give a more detailed description of the model and of the calculations that lead to the various formulas presented in the paper.
(PDF)
