# Correction: Fluctuations, Correlations and the Estimation of Concentrations inside Cells

**DOI:** 10.1371/journal.pone.0192091

**Published:** 2018-01-25

**Authors:** Emiliano Pérez Ipiña, Silvina Ponce Dawson

There is a notation error in the sixth sentence of the fourth paragraph in the Results section. The correct sentence reads as follows: We may then consider that, after a time, *T*_*obs*_, there is only an approximation to *p*_*b*_ given by ${\tilde{p}}_{b}={\bar{N}}^{\left(f\right)}(T_{obs})/({\bar{N}}^{\left(f\right)}(T_{obs})+K_{D}V_{obs})$ and that fluctuations in *N*^(*f*)^, which decay with their own correlation times, impact directly on var(*N*^(*b*)^) (and on $\mathrm{var}({\bar{N}}^{\left(b\right)})$).

There is an error in S1 Text. Eq 13 has a notation problem. Please view the correct S1 Text below.

## Supporting information

S1 Text Model and calculations. In this text we give a more detailed description of the model and of the calculations that lead to the various formulas presented in the paper.
(PDF)
