# Supplementary material for: Fluctuations, Correlations and the Estimation of Concentrations inside Cells
Source: PLoS One. 2016 Mar 10;11(3):e0151132. doi: 10.1371/journal.pone.0151132 (PMC4786111; doi:10.1371/journal.pone.0151132)
Supplement: S1 Text — In this text we give a more detailed description of the model and of the calculations that lead to the various formulas presented in the paper. (PDF) [file pone.0151132.s001.pdf]

Supplementary Information for "Fluctuations, correlations and the estimation of concentrations inside cells"

Emiliano Pérez Ipiña, Silvina Ponce Dawson*
* silvina@df.uba.ar

1 The model

We consider a system of particles (*e.g.*, transcription factors or substrate molecules), $P^{(f)}$, that diffuse with (free) coefficient, D_f, and react with binding sites, S, according to [1–3]:

$$P^{(f)} + S \underset{k_{off}}{\overset{k_{on}}{\rightleftarrows}} P^{(b)}. \quad (1)$$

We assume that the binding sites diffuse with coefficient $D_S \ll D_f$ (in all the examples that we analyze we use $D_S = 0$) and that S is so massive that the free coefficient of $P^{(b)}$ is D_S too. We consider a total volume, V_T, over which the molecules diffuse and the concentrations, $[P^{(f)}]$, $[P^{(b)}]$, $[S]$, are approximately constant, uniform and in equilibrium among themselves so that they satisfy:

$$\begin{aligned} [P^{(f)}][S] &= K_D[P^{(b)}], \\ [P^{(b)}] &= \frac{[P^{(f)}][S_T]}{K_D + [P^{(f)}]}, \\ [S_T] \equiv [P^{(b)}] &+ [S], \end{aligned} \quad (2)$$

with $[S_T]$ the total concentration of binding sites and $K_D \equiv k_{off}/k_{on}$ the dissociation constant of the reaction. We consider an observation volume, V_{obs}, inside V_T, where the number of molecules, $N^{(f)}$, $N^{(b)}$ and $N^{(S)}$, are counted. The means of these stochastic variables satisfy $\langle N^{(f)}\rangle = [P^{(f)}]V_{obs}$, $\langle N^{(b)}\rangle = [P^{(b)}]V_{obs}$ and $\langle N^{(S)}\rangle = [S]V_{obs}$ if $D_s \neq 0$. If $D_S = 0$ and $V_{obs} \ll V_T$, there could be a local equilibrium in V_{obs} slightly different from the one in V_T that depends on the (fixed) total number of binding sites in V_{obs}, $N_{ST} \equiv N^{(b)} + N^{(S)}$.

1.1 Variance and relative errors

The aim is to determine the difference between the mean, $\langle N^{(s)}\rangle$, and the average, $\overline{N}^{(s)}(T_{obs})$, of each stochastic variable, $N^{(s)}$, $s = f, b, S$, after an observation time, T_{obs} which is given by:

$$\overline{N}^{(s)}(T_{obs}) = \frac{1}{T_{obs}} \int_0^{T_{obs}} dt N^{(s)}. \quad (3)$$

We estimate these differences $\overline{N}^{(s)} - \langle N^{(s)}\rangle$ *via* the variance of the average:

$$\text{var}\left(\overline{N}^{(s)}(T_{obs})\right) \equiv \langle\left(\overline{N}^{(s)}(T_{obs}) - \langle N^{(s)}\rangle\right)^2\rangle, \quad (4)$$

which we use to compute the relative error as:

$$\Delta_r(\overline{N}^{(s)}) \equiv \left(\text{var}\left(\overline{N}^{(s)}(T_{obs})\right)\right)^{1/2} / \langle N^{(s)}\rangle. \quad (5)$$

These are the extension to the case with more than one binding site of the equations presented in the main body of the paper. The variance of the average is given by:

$$\begin{aligned} \mathrm{var}\left(\overline{N}^{(s)}(T_{obs})\right) &= \mathrm{var}\left(\frac{1}{T_{obs}}\int_0^{T_{obs}} dt N^{(s)}(t)\right) \\ &= \frac{1}{T_{obs}^2}\langle \int_0^{T_{obs}} dt' \int_0^{T_{obs}} dt \left(N^{(s)}(t)-\langle N^{(s)}\rangle\right)\left(N^{(s)}(t')-\langle N^{(s)}\rangle\right)\rangle \qquad (6) \\ &= \frac{1}{T_{obs}^2}\int_0^{T_{obs}} dt' \int_0^{T_{obs}} dt \langle \left(N^{(s)}(t)-\langle N^{(s)}\rangle\right)\left(N^{(s)}(t')-\langle N^{(s)}\rangle\right)\rangle. \qquad (7) \end{aligned}$$

Thus, it is related [4] to the autocorrelation function (ACF),

$$G^{(s)}(\tau) = \langle (N^{(s)}(t)-\langle N^{(s)}\rangle)(N^{(b)}(t+\tau)-\langle N^{(s)}\rangle)\rangle, \qquad (8)$$

by:

$$\mathrm{var}\left(\overline{N}^{(s)}(T_{obs})\right) = \frac{1}{T_{obs}^2}\int_0^{T_{obs}} dt' \int_0^{T_{obs}} dt G^{(s)}(t'-t). \qquad (9)$$

In order to compute $\Delta_r(\overline{N}^{(s)})$ we then need to compute the integral of the ACF.

1.2 Auto-Correlation Function

For the analytic calculations we compute the ACF as in the case of FCS experiments [2,3,5]. Namely, instead of adding all the particles of species (s) in V_{obs} to compute $N^{(s)}$, we add all the particles of species (s) in V_T but with a Gaussian weight: $N^{(s)} = \int_{V_T} d^3\vec{r} I(\vec{r}) c^{(s)}$ where $I(\vec{r}) = \exp\left(-\frac{r^2}{2a^2}\right)$, $r = |\vec{r}|$, a is half the waist of the Gaussian and $c^{(s)} = \sum_{i_s} \delta(\vec{r}-\vec{r}_{i_s}(t))$ with the sum running over all the molecules of species (s) and $\vec{r}_{i_s}(t)$ the location of each of them at time t. In this way, it is $V_{obs} = \int d^3\vec{r} I(\vec{r}) = 8\pi^{3/2}a^3$ and:

$$G^{(s)}(\tau) = \int d\vec{r} \int d\vec{r}\,' \langle \delta c^{(s)}(\vec{r},0)\delta c^{(s)}(\vec{r}\,',\tau)\rangle, \qquad (10)$$

where $\delta c^{(s)} \equiv c^{(s)}(\vec{r},t) - \langle N^{(s)}\rangle / V_{obs}$. As done in [5,6], for the analytic computation of $G^{(s)}(\tau)$ we calculate the differences, $\delta c^{(s)}$, for the 3 species of the system, as the solution of the reaction-diffusion equations that describe the dynamics of the concentrations of $P^{(f)}$, $P^{(b)}$ and S, linearized around the equilibrium of Eqs. (2). In the case of immobile binding sites ($D_S = 0$), it suffices to consider the equations for $P^{(f)}$ and $P^{(b)}$ only. From now we will restrict all calculations to this case. The $D_S \neq 0$ can be handled similarly [6]. The reaction-diffusion equations then read:

$$\begin{aligned} \frac{\partial P^{(f)}}{\partial t} &= D_f \nabla^2 P^{(f)} - k_{on} P^{(f)}([S_T]-P^{(b)}) + k_{off} P^{(b)}, \\ \frac{\partial P^{(b)}}{\partial t} &= k_{on} P^{(f)}([S_T]-P^{(b)}) - k_{off} P^{(b)}, \end{aligned} \qquad (11)$$

where we use $P^{(f)}$ and $P^{(b)}$ to denote the concentrations in a generic case. These equations linearized around the equilibrium solution, Eqs. (2), read:

$$\begin{aligned} \frac{\partial \delta c^{(f)}}{\partial t} &= D_f \nabla^2 \delta c^{(f)} - k_{on}([S_T]-[P^{(b)}])\delta c^{(f)} \\ &\quad +(k_{off}+k_{on}[P^{(f)}])\delta c^{(b)}, \\ \frac{\partial \delta c^{(b)}}{\partial t} &= k_{on}([S_T]-[P^{(b)}])\delta c^{(f)} - (k_{off}+k_{on}[P^{(f)}])\delta c^{(b)}. \end{aligned} \qquad (12)$$

An alternative linearization is obtained if we assume that $P^{(f)}$ approaches its equilibrium solution much faster than $P^{(b)}$ and replace Eqs. (11) by the following linear system:

$$\begin{aligned}\frac{\partial P^{(f)}}{\partial t} &= D_f\nabla^2 P^{(f)} - k_{on}[S_T]P^{(f)} + (k_{off} + k_{on}P^{(f)})\delta c^{(b)},\\ \frac{\partial \delta c^{(b)}}{\partial t} &= k_{on}[S_T]P^{(f)} - (k_{off} + k_{on}P^{(f)})\delta c^{(b)}.\end{aligned} \tag{13}$$

Clearly $\delta c^{(f)}$ satisfies the same equation as $P^{(f)}$ in this case. We define $p_b \equiv [P^{(b)}]/[S_T] = \langle N^{(b)}\rangle/N_{ST}$ as the equilibrium probability that the sites be bound.

In order to obtain the correlation times we express Eq. (10) in terms of the (branches of) eigenvalues and eigenvectors of Eqs. (12) or (13) as done in [5]:

$$G^{(s)}(\tau) = \frac{1}{(2\pi)^3}\int d\vec{\xi}\left(\hat{I}(\vec{\xi})\right)^2 \sum_m X_j^{(m)} \exp(\lambda^{(m)}\tau)(X^{-1}\sigma^2)_j^{(m)} \tag{14}$$

where the subscript, j, refers to the species ($j = 1$ for $s = f$, and $j = 2$ for $s = b$) and the index, (m), labels the eigenvalues, $\hat{I}(\vec{\xi})$ is the Fourier transform of $I(\vec{r})$ and $\vec{\xi}$ is the conjugate variable of $\vec{r}$, X is the matrix of eigenvectors, $\lambda^{(m)}$ is the m-th eigenvalue and σ^2 is the matrix of initial correlations between the species, $\sigma^2_{ij} = \langle\delta N^{(s)}(0)\delta N^{(s')}(0)\rangle$ with i, j the indices corresponding to species s and s', respectively. As in [6] we assume that $\langle\delta c^{(s)}(\vec{r}, t)\delta c^{(s')}(\vec{r}', t)\rangle = \mathrm{var}(N^{(s)})/V_{obs}\delta_{ij}\delta(\vec{r} - \vec{r}')$ with a Poisson statistics for $s = f$, $\mathrm{var}(N^{(f)}) = \langle N^{(f)}\rangle$, and binomial for $s = b$, $\mathrm{var}(N^{(b)}) = (1 - p_b)\langle N^{(b)}\rangle$. The branches of eigenvalues in Fourier space of Eqs. (12) or (13) can be written as:

$$\lambda^{(1,2)} = -\frac{1}{2}\left(k_{off} + k_{on}(\tilde{S} + [P^{(f)}]) + D_f\xi^2 \pm \sqrt{\rho}\right), \tag{15}$$

where $\rho = (k_{off} + k_{on}(\tilde{S} + [P^{(f)}]))^2 + 2D_f\xi^2(k_{on}\tilde{S} - k_{on}[P^{(f)}] - k_{off}) + D_f^2\xi^4$, with $\tilde{S} = [S_T]$ in the case of Eqs. (13) and $\tilde{S} = [S] = [S_T] - [P^{(b)}]$ in the case of Eqs. (12). The matrix of eigenvectors can then be written as:

$$X = \begin{bmatrix} [P^{(f)}] + K_D - \tilde{S} - \frac{D_f}{k_{on}}\xi^2 + \frac{\sqrt{\rho}}{k_{on}} & [P^{(f)}] + K_D - \tilde{S} - \frac{D_f}{k_{on}}\xi^2 - \frac{\sqrt{\rho}}{k_{on}} \\ 2\tilde{S} & 2\tilde{S} \end{bmatrix}, \tag{16}$$

The mean (asymptotic) correlation time of the bound particles (or occupied sites) defined as [4]:

$$\tau^{(b)} \equiv \frac{1}{\mathrm{var}(N^{(b)})}\int_0^\infty d\tau G^{(b)}(\tau), \tag{17}$$

can be computed exactly. In the case of Eqs. (13) it is:

$$\tau^{(b)} = \frac{p_b N_{ST}}{\sqrt{2\pi^3}a[P^{(f)}]D_f} + \frac{1 - p_b}{k_{off}}, \tag{18}$$

and in the case of Eqs. (12) it is:

$$\tau^{(b)} = \frac{p_b(1 - p_b)N_{ST}}{\sqrt{2\pi^3}a[P^{(f)}]D_f} + \frac{1 - p_b}{k_{off}}. \tag{19}$$

The asymptotic time (19) is similar to the one obtained in [7] and that of Eq. (18) to the one obtained in [8], the only differences being due to geometric factors.

1.3 Approximated ACF in two limits.

As in [3] there are two limits for which the eigenvalue branches can be expressed as $\lambda^{(i)} = -\nu_i$ or as $-D_i\xi^2$ with ν_i a function of the reaction rates and concentrations and D_i depending, in general, on these quantities and on D_f. These are the *fast reaction* (*fr*) and the *fast diffusion* limits (*fd*) defined by $\tau_r \ll \tau_f$ and $\tau_f \ll \tau_r$, respectively with τ_f the diffusion timescale and τ_r the reaction one:

$$\tau_f \equiv a^2 D_f^{-1}, \quad \tau_r \equiv (k_{off} + k_{on}\tilde{S} + k_{on}[P^{(f)}])^{-1}, \tag{20}$$

where $\tilde{S} = [S_T]$ in the case of Eqs. (13) and $\tilde{S} = [S]$ in the case of Eqs. (12). Given Eqs. (18)–(19) these inequalities imply that one of the two terms prevails in the sum that defines $\tau^{(b)}$ in each of these limits.

The *fr* limit is obtained by expanding the eigenvalues and eigenvectors in powers of the wavenumber, ξ and keep the terms in Eq. (14) up to order ξ^2. In this way the eigenvalues, Eqs. (15), are approximately given by:

$$\lambda^{(1)} \approx -D_f\xi^2/(1+\beta) \tag{21}$$

$$\lambda^{(2)} \approx -(k_{off}(1+\beta)/(1-p_b)) - \beta D_f\xi^2/(1+\beta) \tag{22}$$

with $\beta = p_b N_{ST}/\langle N^{(f)}\rangle$ if we use Eqs. (13) and $\beta = p_b(1-p_b)[S_T]/[P^{(f)}]$. Eq. (21) prescribes a diffusive behavior with an effective diffusion coefficient, $D_{ef} \equiv D_f/(1+\beta)$, that coincides with the collective one [1]:

$$D_{coll} \equiv \frac{D_f}{1 + \frac{[S]^2}{[S_T]k_D}}, \tag{23}$$

if we use the linearization of Eqs. (12) and the single molecule one:

$$D_{sm} \equiv \frac{D_f}{1 + \frac{[S]}{K_D}}, \tag{24}$$

if we use the linearization of Eqs. (13). Eq. (22) also gives rise to an exponential decay in time. Furthermore, under the assumption that $\tau_r \ll \tau_f$ it can be shown that $\lambda^{(2)} \approx -(k_{off}(1+\beta)/(1-p_b))$, *i.e.*, the exponential decay prevails. Thus, one of the timescales is purely diffusive and the other is reaction-dominated in this limit. It is worth mentioning that, for long enough time lags, τ, the $\xi \sim 0$ expansion is always valid. This is a consequence of diffusion not being characterized by a single time-scale, but by a function of timescales that increase with the wavelength, $1/\xi$. The *fr* limit holds for all time lags when even the shortest relevant diffusion timescale, $\sim a^2/D_f$, is larger than the reaction one. The *fd* limit is exactly opposite to the *fr* one and, in practice, can be obtained by taking the limit as $\xi \to \infty$ in the eigenvalues and eigenvectors. In this case the eigenvalues are approximately given by:

$$\lambda^{(1)} \approx -D_f\xi^2 \tag{25}$$

$$\lambda^{(2)} \approx -\frac{k_{off}}{1-p_b} \tag{26}$$

Also in this case one time-scale is purely diffusive (with the freee diffusion coefficient, D_f) and the other is reaction-dominated.

Under the *fr* or the *fd* approximations each eigenvalue branch is characterized by a single type of time-scale that is either purely diffusive or exponentially decaying (reaction-dominated). The terms with these types of time-dependence can readily be integrated using Eq. (14) giving rise to terms in the ACF that scale with the lag time, τ, as $1/(1+\tau/\tau_{Di})^{3/2}$ and $\exp(-\nu_i\tau)$, respectively. Thus, in both these limits the ACFs can be written as:

$$G^{(s)}(\tau) = \sum_{i=1}^{2} G_i^{(s)}(\tau) = \sum_{i=1}^{2}, \frac{G_{oi}^{(s)}}{\left(1+\frac{\tau}{\tau_{Di}}\right)^{3/2}} e^{-\nu_i\tau}, \tag{27}$$

where the quantities $\tau_{Di} = a^2/D_i$ represent the diffusive time-scales with D_i the corresponding diffusion coefficient and ν_i the inverse of the reaction-dominated time-scale. As just discussed, in the *fr* and the *fd* limits it is either $\nu_i = 0$ when D_i is finite or $D_i \to \infty$ when $\nu_i \neq 0$ in each of these terms. The weights, $G_{oi}^{(s)}$, are linear combinations of the covariances between the stochastic variables that satisfy $G_o^{(s)} \equiv \sum_i G_{oi}^{(s)} = \mathrm{var}(N^{(s)})$. Inserting the eigenvalues and eigenvectors in the *fd* or *fr* approximations in Eq. (14) as done in [2,3,6] we obtain:

$$G^{(f)}(\tau) = \mathrm{var}(N^{(f)}) \left(1 + \frac{|\tau|}{\tau_f}\right)^{-3/2}, \tag{28}$$

$$G^{(b)}(\tau) = \mathrm{var}(N^{(b)}) e^{-|\tau|/\tau_{off}}, \tag{29}$$

with τ_f defined in Eq. (20) and

$$\tau_{off}^{-1} = \frac{k_{off}}{1 - p_b}, \tag{30}$$

for both Eqs. (13) and (12). In the *fr* limit we obtain:

$$G^{(f)}(\tau) = \mathrm{var}(N^{(f)}) \left(\frac{(1 + \frac{|\tau|}{\tau_{ef}})^{-3/2}}{1+\beta} + \frac{\beta e^{-\frac{|\tau|}{\tilde{\tau}_r}}}{1+\beta} \right) \tag{31}$$

$$G^{(b)}(\tau) = \mathrm{var}(N^{(b)}) \left(\frac{\beta(1 + \frac{|\tau|}{\tau_{ef}})^{-3/2}}{1+\beta} + \frac{e^{-\frac{|\tau|}{\tilde{\tau}_r}}}{1+\beta} \right), \tag{32}$$

with

$$\tau_{ef} \equiv \frac{a^2}{D_{ef}} \equiv \frac{a^2(1+\beta)}{D_f}, \quad \tilde{\tau}_r = \frac{\tau_{off}}{1+\beta} \tag{33}$$

and $\beta = p_b N_{ST}/\langle N^{(f)}\rangle$ if we use Eqs. (13) and $\beta = p_b(1-p_b)N_{ST}/\langle N^{(f)}\rangle$ if we use Eqs. (12).

1.4 Variance and relative errors.

We combine Eqs. (4) and (5) to compute the (square of the) relative error as:

$$\Delta_r(\overline{N}^{(s)})^2 = \frac{\mathrm{var}\left(N^{(s)}\right)}{T_{obs}^2 \langle N^{(s)}\rangle} \int_0^{T_{obs}} dt' \int_0^{T_{obs}} dt G^{(s)}(t'-t). \tag{34}$$

Inserting Eqs. (28), (29), (31) and (32) into Eq. (34) we derive analytic expressions for $\Delta_r(\overline{N}^{(s)})$ in the *fd* and the *fr* limits. We notice that Eqs. (28), (29), (31) and (32) are linear combinations of functions of the form:

$$\Phi_D(\tau) = \left(1 + \frac{|\tau|}{\tau_D}\right)^{-3/2} \& \ \Phi_R(\tau) = \exp\left(-\frac{|\tau|}{\tau_R}\right), \tag{35}$$

that can be easily integrated as prescribed in Eq. (34). In computing these integrals we must recall that the ACFs are even functions of the lag time, τ. We obtain:

$$\left(\Delta_r(\overline{N}^{(f)})\right)^2 = \frac{\mathrm{var}\left(N^{(f)}\right)}{\langle N^{(f)}\rangle^2} \frac{4\tau_f}{T_{obs}} \left(1 + \frac{2\tau_f}{T_{obs}}\left(1 - \left(1 + \frac{T_{obs}}{\tau_f}\right)^{1/2}\right)\right), \tag{36}$$

$$\left(\Delta_r(\overline{N}^{(b)})\right)^2 = \frac{\mathrm{var}\left(N^{(b)}\right)}{\langle N^{(b)}\rangle^2} \frac{2\tau_{off}}{T_{obs}} \left(1 + \frac{\tau_{off}}{T_{obs}}\left(e^{-T_{obs}/\tau_{off}} - 1\right)\right), \tag{37}$$

in the *fd* limit and, in the *fr* one,

$$\left(\Delta_r(\overline{N}^{(f)})\right)^2 = \frac{\mathrm{var}\left(N^{(f)}\right)}{(1+\beta)\langle N^{(f)}\rangle^2} \times$$
$$\left(\frac{2\tilde{\tau}_r\beta}{T_{obs}}\left(1+\frac{\tilde{\tau}_r}{T_{obs}}\left(e^{-T_{obs}/\tilde{\tau}_r}-1\right)\right)+\frac{4\tau_{ef}}{T_{obs}}\left(1+2\frac{\tau_{ef}}{T_{obs}}\left(1-\left(1+\frac{T_{obs}}{\tau_{ef}}\right)^{1/2}\right)\right)\right), \quad (38)$$

$$\left(\Delta_r(\overline{N}^{(b)})\right)^2 = \frac{\mathrm{var}\left(N^{(b)}\right)}{(1+\beta)\langle N^{(b)}\rangle^2} \times$$
$$\left(\frac{2\tilde{\tau}_r}{T_{obs}}\left(1+\frac{\tilde{\tau}_r}{T_{obs}}\left(e^{-T_{obs}/\tilde{\tau}_r}-1\right)\right)+\frac{4\tau_{ef}\beta}{T_{obs}}\left(1+2\frac{\tau_{ef}}{T_{obs}}\left(1-\left(1+\frac{T_{obs}}{\tau_{ef}}\right)^{1/2}\right)\right)\right), \quad (39)$$

For some illustrations we also use simpler expressions of $\Delta_r(\overline{N}^{(s)})$ that provide an easier interpretation in terms of correlation times. Namely, we approximate

$$\Phi_D(\tau) \approx 1 \quad \text{if } |\tau| \leq 2\tau_D, \quad (40)$$
$$\Phi_R(\tau) \approx 1 \quad \text{if } |\tau| \leq \tau_R, \quad (41)$$

and zero otherwise. With these approximations the corresponding mean correlation times remain the same as if we used Eqs. (28)–(29) or (31)–(32) and the relative errors read, in the fast diffusion limit:

$$\left(\Delta_r(\overline{N}^{(f)})\right)^2 = \frac{4\tau_f}{T_{obs}}\left(1-\frac{2\tau_f}{2T_{obs}}\right), \text{ for } T_{obs} \geq 2\tau_f,$$
$$\left(\Delta_r(\overline{N}^{(b)})\right)^2 = \frac{2\tau_{off}}{T_{obs}}\left(1-\frac{\tau_{off}}{2T_{obs}}\right), \text{ for } T_{obs} \geq \tau_{off},$$
$$\left(\Delta_r(\overline{N}^{(f)})\right)^2 = \left(\Delta_r(\overline{N}^{(b)})\right)^2 = 1, \text{ otherwise}, \quad (42)$$

and, in the fast reaction limit,

$$\left(\Delta_r(\overline{N}^{(b)})\right)^2 = \frac{\mathrm{var}\left(N^{(b)}\right)}{(1+\beta)\langle N^{(b)}\rangle^2} \times \quad (43)$$
$$\begin{cases} 1, & \text{if } T_{obs} \leq \tilde{\tau}_r, \\ \left(\frac{2\tilde{\tau}_r}{T_{obs}}\left(1-\frac{\tilde{\tau}_r}{2T_{obs}}\right)+\beta\right), & \text{if } \tilde{\tau}_r \leq T_{obs} \leq 2\tau_{ef}, \\ \left(\frac{2\tilde{\tau}_r}{T_{obs}}\left(1-\frac{\tilde{\tau}_r}{2T_{obs}}\right)+\beta\frac{4\tau_{ef}}{T_{obs}}\left(1-\frac{2\tau_{ef}}{2T_{obs}}\right)\right), & \text{if } T_{obs} \geq 2\tau_{ef}. \end{cases}$$

In some instances we also compute $\Delta_r(\overline{N}^{(s)})^2$ outside the *fd* or the *fr* limits by inserting Eq. (14) into (34) and performing the integral in ξ numerically.

1.5 The fast diffusion and fast reaction limits and the linearization of the reaction-diffusion equations in the case of a single binding site

The case of a single binding site inside a medium with a mean concentration of free particles has been studied using different approaches. In [7] Eqs. (11) were considered with $[S_T]=\delta(\mathbf{r})$ and replacing the second equation of the set by an equation for $p^{(b)}(t)$, the instantaneous probability that the site be occupied. Namely, the evolution equations of [7] are equivalent to:

$$\frac{\partial[P^{(f)}]}{\partial t} = D_f\nabla^2[P^{(f)}]-(k_{on}[P^{(f)}]p_b-k_{off}(1-p_b))\delta(\mathbf{r}),$$
$$\frac{dp_b}{dt} = k_{on}[P^{(f)}](\mathbf{r}=0,t)(1-p_b)-k_{off}p_b. \quad (44)$$

Using this point-like distribution for the binding site leads to unrealistic divergences. The way to correct for them is to work with a "smoothed" version given by Eqs. (11) with $S_T = 3/(4\pi a_s^3)$ for $|\mathbf{r}| \leq a_s$ and zero otherwise and where a_s represents the size of the binding site or the distance over which it interacts with the free particles. The approach of [8], built upon the previous work of [9] is very different. Namely, in [8, 9] it is assumed that the free particles interact with the binding site only when they are at a distance $|\mathbf{r}| = a_s$ from it and that $P^{(f)}$ is only defined for $|\mathbf{r}| \geq a_s$. The initial condition guarantees the spherical symmetry of the problem. Identifying $\langle N^{(f)}(r \geq a_s, t)\rangle = \int_{|\mathbf{r}|\geq a_s} dr 4\pi r^2 [P^{(f)}]$, the description of [8, 9] is equivalent to considering the equations:

$$\frac{\partial[P^{(f)}]}{\partial t} = \frac{D_f}{r^2}\frac{\partial\left(r^2[P^{(f)}]\right)}{\partial r}, \qquad |\mathbf{r}| \geq a \quad \frac{\partial[P^{(f)}]}{\partial r} \to 0 \,\text{for}\, r \to \infty, \tag{45}$$

$$p_b + \int_{|\mathbf{r}|\geq a} dr 4\pi r^2 [P^{(f)}] = constant, \tag{46}$$

$$\frac{dp_b}{dt} = k_{on}(1-p_b)[P^{(f)}](a_s, t) - k_{off} p_b. \tag{47}$$

The conservation of the total number of particles, Eq. (46) implies the boundary condition:

$$4\pi a_s^2 \frac{\partial[P^{(f)}]}{\partial r}|_{r=a} = k_{on}(1-p_b)[P^{(f)}](a_s,t) - k_{off}p_b = k_{on}(1-p_b)[P^{(f)}](a_s,t) - k_{off}(1 - \int_{|\mathbf{r}|\geq a} dr 4\pi r^2 [P^{(f)}]). \tag{48}$$

Both in the approach of [7] and of [8, 9] the dynamic equations are nonlinear. The linearization of either approach is different and is equivalent with having started with Eqs. (12) in the case of [7] and with Eqs. (13) in the case of [8, 9]. Our way to think of this problem is closer to that of [7]. Namely, we consider the reaction-diffusion system given by Eqs. (11) with $P^{(b)} = p_b(t)[S_T]$, $[S] = (1 - p_b(t))[S_T]$, and with a spatially restricted distribution, $[S_T]$, such that $[S_T](r) = 3/4\pi a_s^3$ for $r \leq a_s$ and 0 otherwise. In spite of the non-uniformity of $[S_T]$, the equilibrium solution is spatially uniform for $[P^{(f)}]$ and piece-wise constant for $[P^{(b)}]$ and $[S]$, with the various concentrations related by Eqs. (2) everywhere in space. The number of bound particles (or, equivalently, of bound sites) at time t is $N^{(b)}(t) = \int_0^\infty dr 4\pi r^2 P^{(b)}$, and the corresponding ACF, $G^{(b)}$, has the same expression as before. The finite volume over which $[S_T] \neq 0$ limits the spatial integral that is involved in the computation of $G^{(b)}$. Given that the equilibrium solution and the dynamical equations (11) coincide in the $r \leq a_s$ region with those of a similar problem but where $[S_T] = 3/(4\pi a_s^3)$ everywhere in space the way in which we decided to handle this problem is to use the solution of the latter and then restrict the spatial integral in Eq. (10) to the $r \leq a_s$ region (where $[S_T]$ is uniform in our current problem). This approximation will be valid as long as the difference between performing the integral in Eq. (10) over all space or over the $r \leq a_s$ be negligible. This means that the characteristic length of the observation volume in this case is of the order of the interaction distance or typical binding site size, *i.e.* $a \sim a_s$. Under this assumption, we can then proceed as described in the previous subsections and as done in [3, 5, 6], linearize the equations and write the ACF in terms of the eigenvalues and eigenvectors of the linear problem. Following these steps, when we compute the asymptotic time, $\tau^{(b)}$, given by Eq. (17) we recover the results of [7] if we use the linearization given by Eqs. (12) and those of [8, 9] if we use the linearization of Eqs. (12) instead. This gives us a reassurance of the validity of our approach. The linearization inside the small volume, $V_o \sim a^3$, is a delicate issue that we will study in more detail in the future. What we have done in this paper is using one or the other linearization in most cases and comparing the predictions of both. Analyzing the *fd* or *fr* limits of the ACFs obtained using one or the other linearization we observe that they are equal in the former and differ in the diffusive time-scale term in the latter. As discussed in the main body of the paper a comparison between the theoretical predictions and the particle simulations shows that the linearization given by Eqs. (12) prescribes the correct correlation times of $G^{(b)}$ when there are several binding sites ($N_{ST} > 1$).

Regardless of the linearization that is used to compute the ACFs, the question then arises as to whether in the small volume, $V_o \sim a^3$, around a single binding site the correct approximation to $G^{(b)}$

and $G^{(f)}$ is the one given by the *fd* or the *fr* limits. Namely, in a generic case with spatially uniform equilibrium concentrations, as the observation volume, $V_o \sim a^3$, goes to zero the diffusive timescale, $\tau_f \sim w_r^2/D_f$, goes to zero as well, τ_r remains finite and the *fd* limit is approached. If V_o is such that it always encloses a binding site and eventually encloses only one we are in the situation described in the previous paragraph. Let us assume, as before, that a is the interaction distance between the site and a free particle so that we can work with $[S_T] = 3/4\pi a^3$. Furthermore, if we are interested in fluctuations in the occupation state of the binding site we cannot go all the way down to zero with V_o, we are basically limited to consider $V_o \sim a^3$. The fact that $[S_T] \sim 1/a^3$ on the other hand implies that $\tilde{S} \propto [S_T]$ can be very large and, given Eqs. (20), τ_r be very small. If the term proportional to $\tilde{S}$ dominates the reaction time-scale we have $\tau_r \propto a^3/k_{on}$. So, in this case, the *fd* limit ($\tau_f \ll \tau_r$) could still hold if $aD_f/k_{on} \gg 1$. Considering the range $a \sim 4 - 40nm$ and $D_f \approx 20\mu m2/s$ (our estimate for the free diffusion coefficient of the transcription factor, Bicoid) this implies that $k_{on} \ll 50 - 500\mu M^{-1}s^{-1}$ for the *fd* limit to hold also when $V_o \sim a^3$ and a single binding site is enclosed. On rates of the order of tens or less $\mu M^{-1}s^{-1}$ are common for protein-protein interactions [10]. In fact, the analysis of the dwell-time distribution between successive bindings of an individual enzyme molecule that we present in the main body of the paper is consistent with a transition from the *fd* to the *fr* limit as the concentration of free particles (in this case, substrate molecules) is increased. In any case, which limit better describes the correlation times in this type of situation should be assessed on a one to one basis. The transition from a situation in which a description in terms of concentrations is valid to one in which it is necessary to deal with individual molecules is another delicate issue that we are planning to study with more detail in the future.

References

1. Pando B, Dawson SP, Mak DOD, Pearson JE. Messages diffuse faster than messengers. Proc Natl Acad Sci (USA). 2006;103(14):5338–5342.

2. Sigaut L, Ponce ML, Colman-Lerner A, Dawson SP. Optical techniques provide information on various effective diffusion coefficients in the presence of traps. Phys Rev E. 2010 Nov;82:051912.

3. Ipiña EP, Dawson SP. From free to effective diffusion coefficients in fluorescence correlation spectroscopy experiments. Phys Rev E. 2013 Feb;87:022706.

4. Berg HC, Purcell EM. Physics of chemoreception. Biophysical Journal. 1977;20(2):193 – 219.

5. Krichevsky O, Bonnet G. Fluorescence correlation spectroscopy: the technique and its applications. Rep Prog Phys. 2002;65(2):251.

6. Pérez Ipiña E, Ponce Dawson S. How long should a system be observed to obtain reliable concentration estimates from the measurement of fluctuations? Biophysical J. 2014;107(11):2674–2683.

7. Bialek W, Setayeshgar S. Physical limits to biochemical signaling. Proc Natl Acad Sci (USA). 2005;102(29):10040–10045.

8. Kaizu K, de Ronde W, Paijmans J, Takahashi K, Tostevin F, ten Wolde PR. The Berg-Purcell Limit Revisited. Biophysical Journal. 2014 Feb;106(4):976–985.

9. Agmon N, Szabo A. Theory of reversible diffusion influenced reactions. The Journal of Chemical Physics. 1990 May;92(9):5270–5284.

10. Dogan J, Jonasson J, Andersson E, Jemth P. Binding Rate Constants Reveal Distinct Features of Disordered Protein Domains. Biochemistry. 2015;54(30):4741–4750.
